# Correction: Endocytosed β2-Microglobulin Amyloid Fibrils Induce Necrosis and Apoptosis of Rabbit Synovial Fibroblasts by Disrupting Endosomal/Lysosomal Membranes: A Novel Mechanism on the Cytotoxicity of Amyloid Fibrils

**DOI:** 10.1371/journal.pone.0141631

**Published:** 2015-10-27

**Authors:** The PLOS ONE Staff

## Notice of Republication

This article was republished on October 9, 2015, to correct an error in the title and citation: “2-Microglobulin” was corrected to “β2-Microglobulin.” The publisher apologizes for the error. Please download this article again to view the correct version. The originally published, uncorrected article and the republished, corrected article are provided here for reference.

## Supporting Information

S1 File Originally published, uncorrected article. (PDF)

S2 File Republished, corrected article. (PDF)
