# Supplementary figures and images for: Endocytosed β2-Microglobulin Amyloid Fibrils Induce Necrosis and Apoptosis of Rabbit Synovial Fibroblasts by Disrupting Endosomal/Lysosomal Membranes: A Novel Mechanism on the Cytotoxicity of Amyloid Fibrils
Source: PLoS One. 2015 Sep 30;10(9):e0139330. doi: 10.1371/journal.pone.0139330 (PMC4589361; doi:10.1371/journal.pone.0139330)

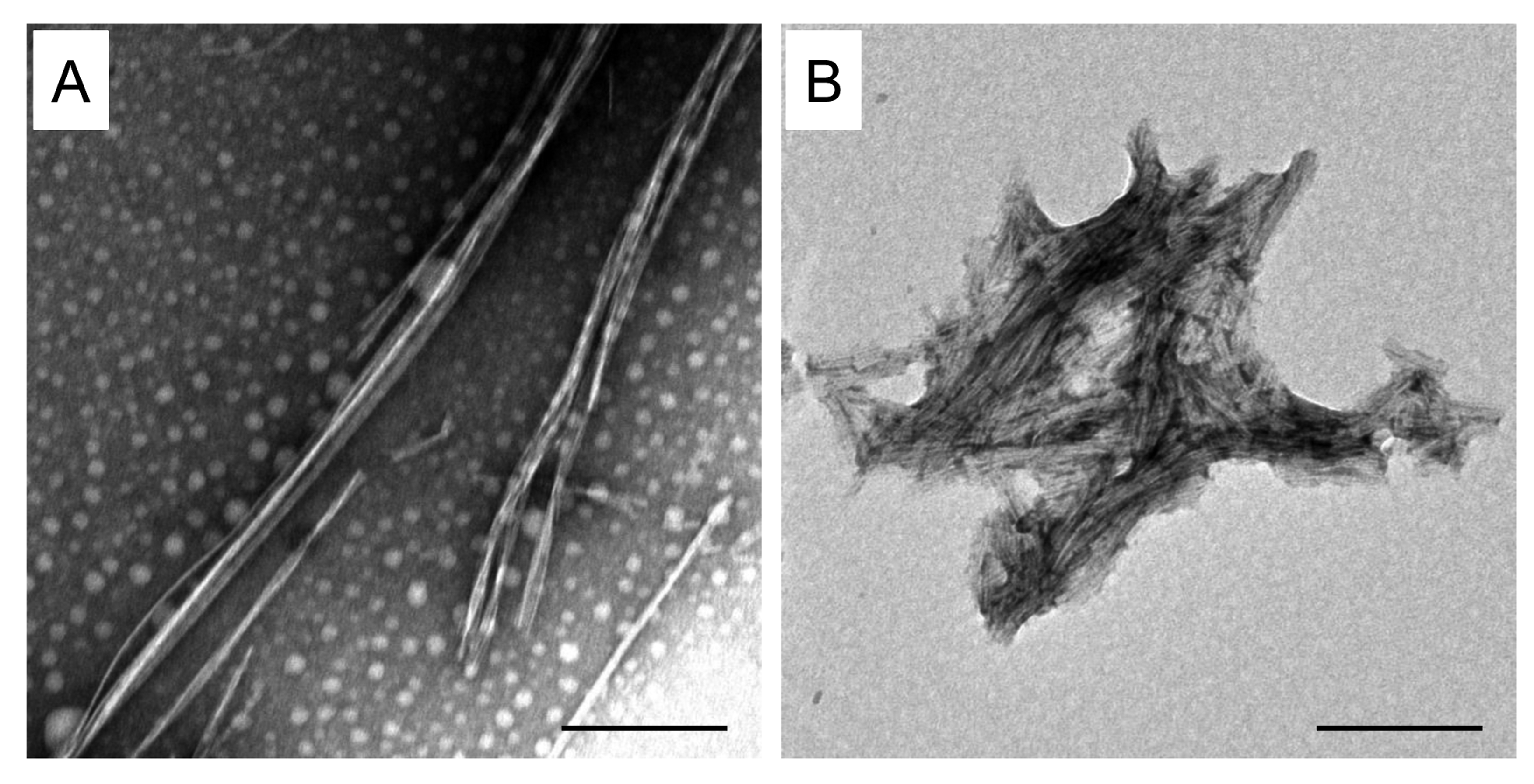

Supplement: S1 Fig — Samples were spread on carbon-coated grids and negatively stained with 1% phosphotungstic acid (pH 7.0). The images were digitally taken with Hitachi H-7650 transmission electron microscope with an acceleration voltage of 80 kV. The scale bars are 200 nm long. (TIF) [file pone.0139330.s001.tif]

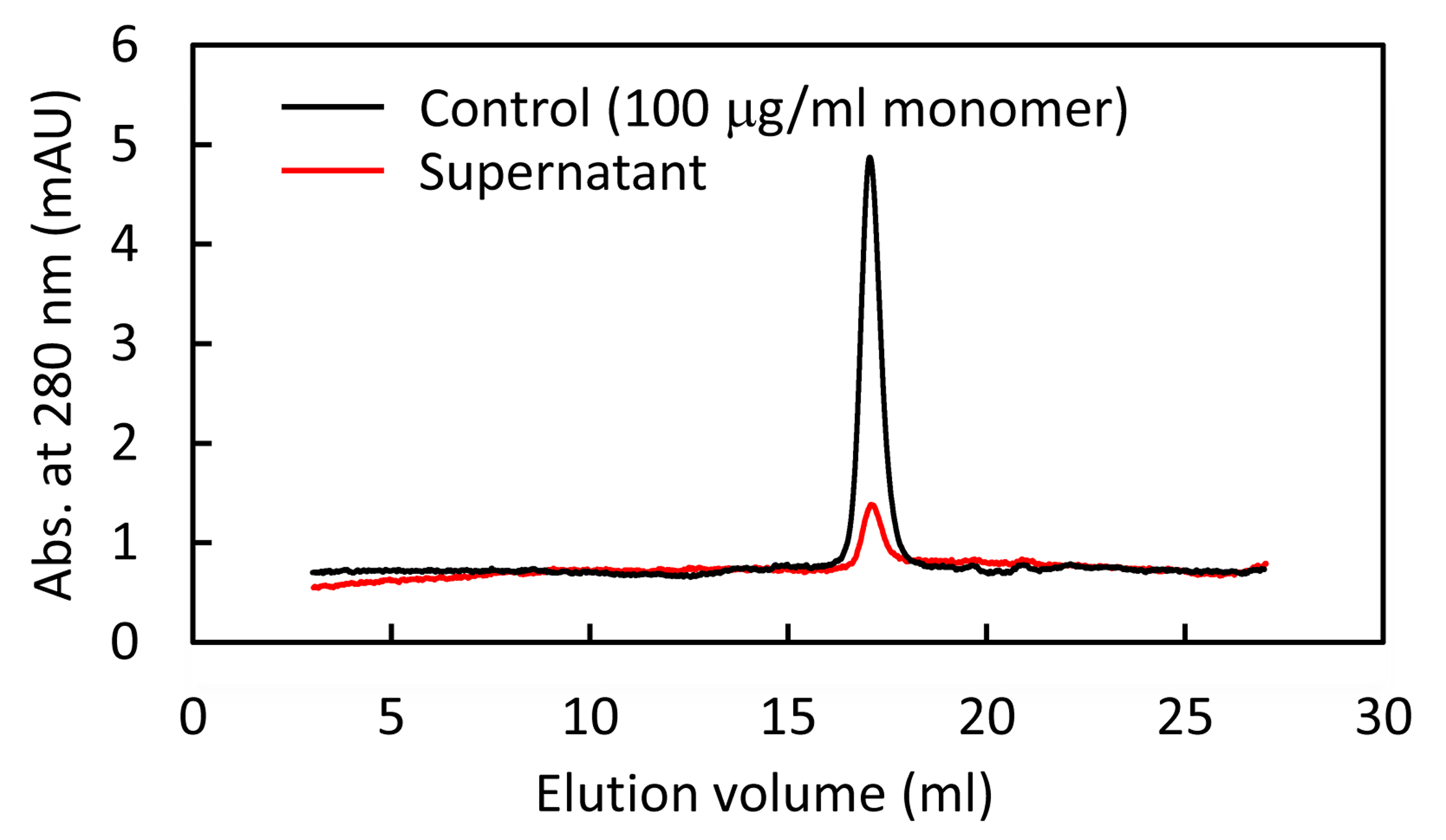

Supplement: S2 Fig — Aliquot of the final fibril preparation was centrifuged at 15 000 rpm for 90 min. A hundred microliters of the supernatant was then applied onto a Superose 12 10/300GL gel-filtration column (GE healthcare, Tokyo, Japan) equilibrated with PBS (-) at 15°C, using a flow rate of 0.5 ml/min while monitoring the absorbance at 280 nm. As a control, 100 μg/ml β2-m monomer solution with 150 mM NaCl was applied. (TIF) [file pone.0139330.s002.tif]

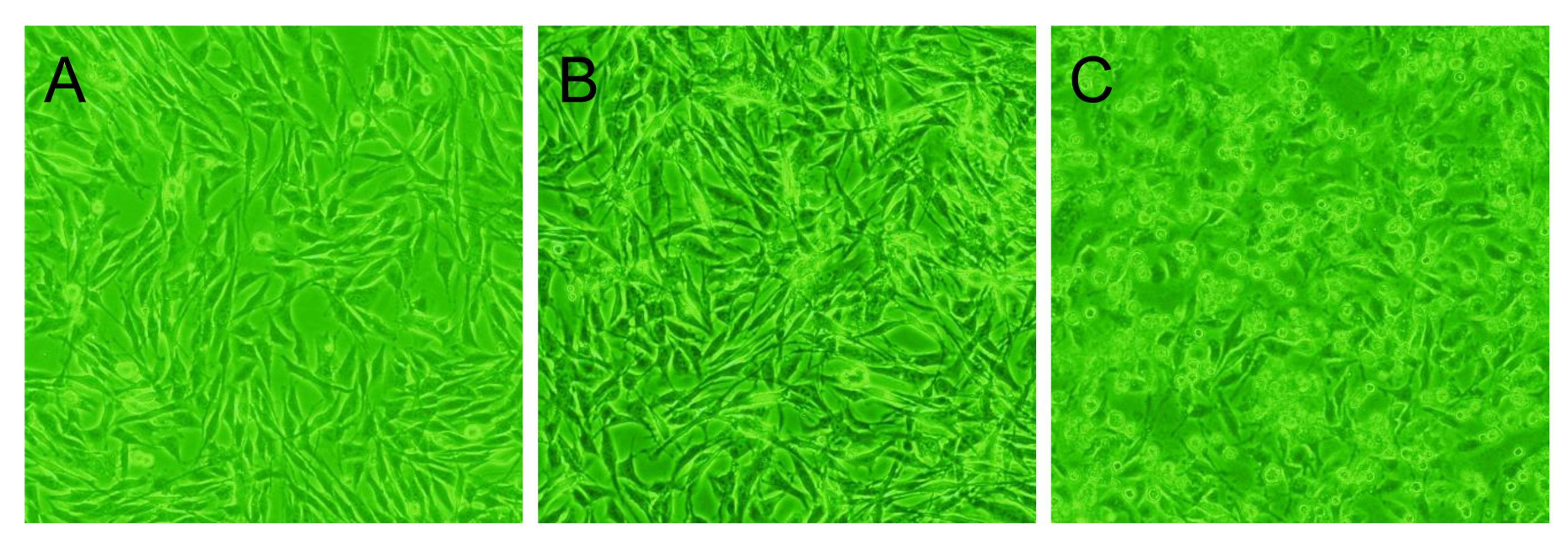

Supplement: S3 Fig — The original magnification was x100. (TIF) [file pone.0139330.s003.tif]

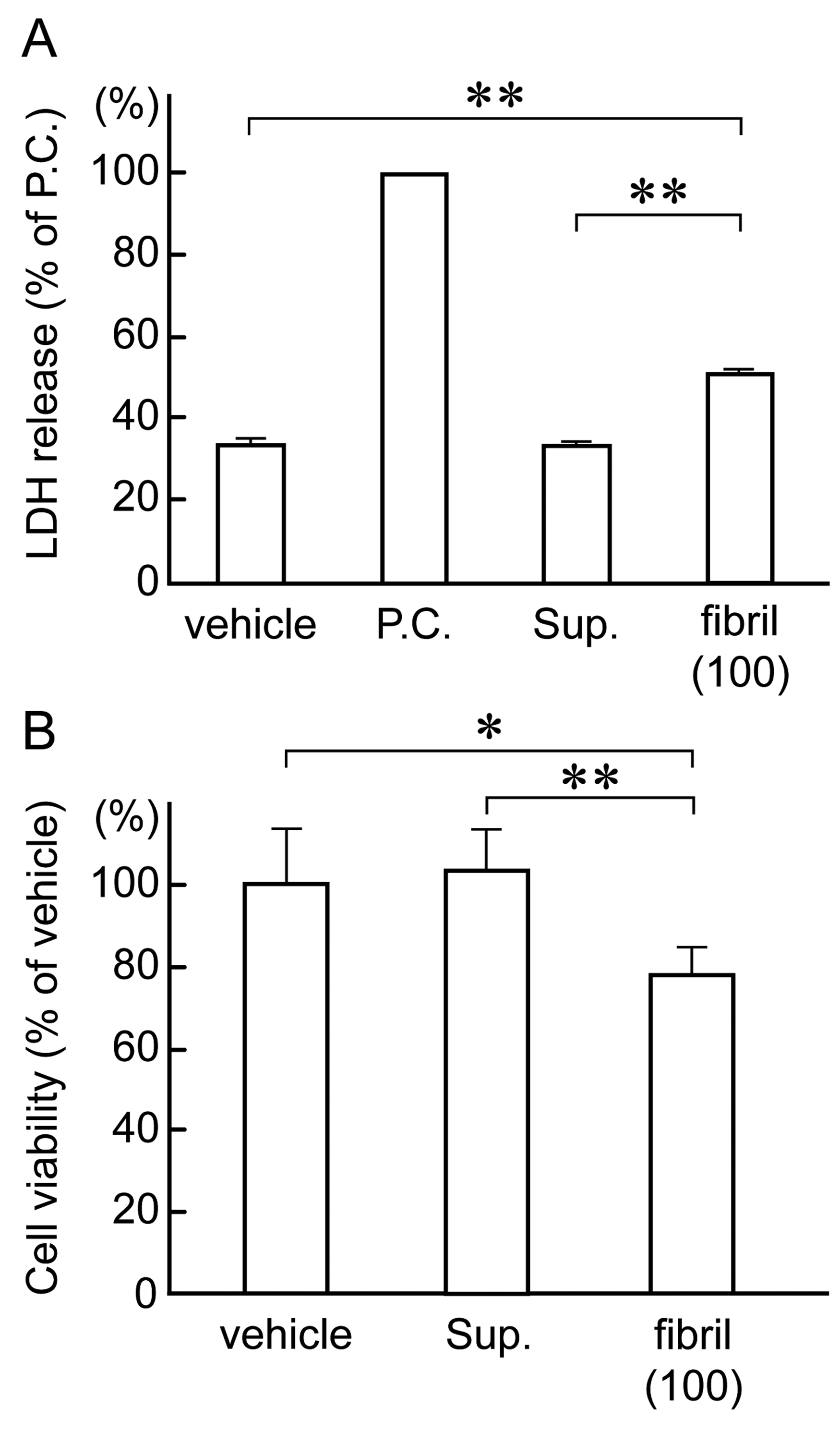

Supplement: S4 Fig — After HIG-82 cells were incubated for 2 days with Ham’s F12 medium containing vehicle buffer, 100 μg/ml β2-m fibrils or the same volume of the supernatant of the fibril preparation, LDH releasing assay (A) and MTT reduction assay (B) were performed as described in Materials and Methods. Data normalized to positive control and vehicle in LDH releasing assay and MTT reduction assay, respectively were presented as mean ± SD of three independent experiments. Statistical analysis was performed by Student’s unpaired t-test. *P < 0.01, **P < 0.001. (TIF) [file pone.0139330.s004.tif]

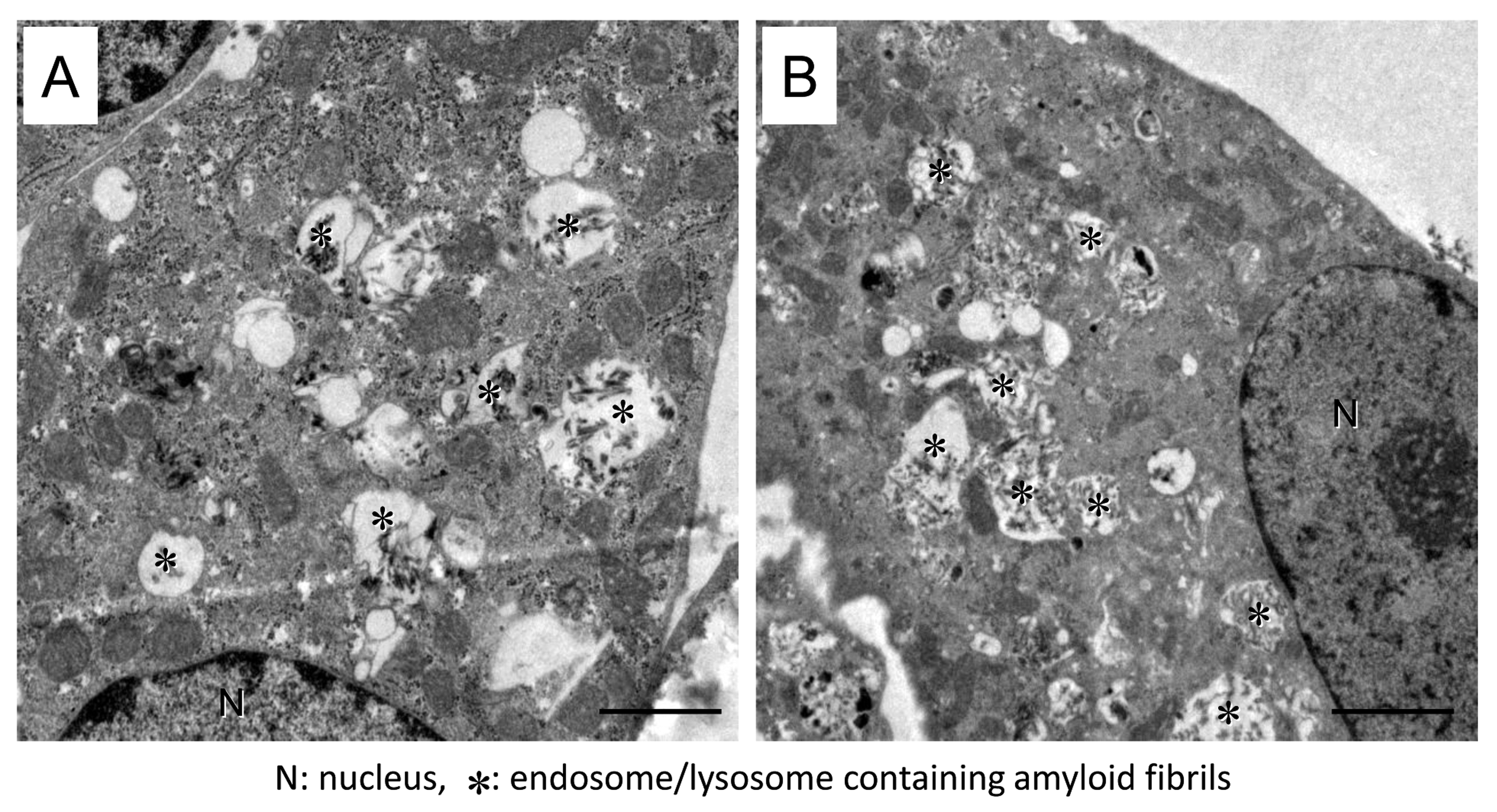

Supplement: S5 Fig — The scale bars are 2 μm long. (TIF) [file pone.0139330.s005.tif]
