# Supplementary material for: Correction: Endocytosed β2-Microglobulin Amyloid Fibrils Induce Necrosis and Apoptosis of Rabbit Synovial Fibroblasts by Disrupting Endosomal/Lysosomal Membranes: A Novel Mechanism on the Cytotoxicity of Amyloid Fibrils
Source: PLoS One. 2015 Oct 27;10(10):e0141631. doi: 10.1371/journal.pone.0141631 (PMC4624719; doi:10.1371/journal.pone.0141631)
Supplement: S1 File — (PDF) [file pone.0141631.s001.pdf]

PLOS ONE

RESEARCH ARTICLE

Endocytosed 2-Microglobulin Amyloid Fibrils Induce Necrosis and Apoptosis of Rabbit Synovial Fibroblasts by Disrupting Endosomal/Lysosomal Membranes: A Novel Mechanism on the Cytotoxicity of Amyloid Fibrils

Tadakazu Okoshi, Itaru Yamaguchi, Daisaku Ozawa, Kazuhiro Hasegawa, Hironobu Naiki*

Division of Molecular Pathology, Department of Pathological Sciences, Faculty of Medical Sciences, University of Fukui, Fukui, Japan

* naiki@u-fukui.ac.jp

Abstract

Dialysis-related amyloidosis is a major complication in long-term hemodialysis patients. In dialysis-related amyloidosis, β2-microglobulin (β2-m) amyloid fibrils deposit in the osteoarticular tissue, leading to carpal tunnel syndrome and destructive arthropathy with cystic bone lesions, but the mechanism by which these amyloid fibrils destruct bone and joint tissue is not fully understood. In this study, we assessed the cytotoxic effect of β2-m amyloid fibrils on the cultured rabbit synovial fibroblasts. Under light microscopy, the cells treated with amyloid fibrils exhibited both necrotic and apoptotic changes, while the cells treated with β2-m monomers and vehicle buffer exhibited no morphological changes. As compared to β2-m monomers and vehicle buffer, β2-m amyloid fibrils significantly reduced cellular viability as measured by the lactate dehydrogenase release assay and the 3-(4,5-di-methylthiazol-2-yl)-2,5-diphenyltetrazolium bromide reduction assay and significantly increased the percentage of apoptotic cells as measured by the terminal deoxynucleotidyl transferase-mediated dUTP nick end labeling method. β2-m amyloid fibrils added to the medium adhered to cell surfaces, but did not disrupt artificial plasma membranes as measured by the liposome dye release assay. Interestingly, when the cells were incubated with amyloid fibrils for several hours, many endosomes/lysosomes filled with amyloid fibrils were observed under confocal laser microscopy and electron microscopy, Moreover, some endosomal/lysosomal membranes were disrupted by intravesicular fibrils, leading to the leakage of the fibrils into the cytosol and adjacent to mitochondria. Inhibition of actin-dependent endocytosis by cytochalasin D attenuated the toxicity of amyloid fibrils. These results suggest that endocytosed β2-m amyloid fibrils induce necrosis and apoptosis by disrupting endosomal/lysosomal membranes, and this novel mechanism on the cytotoxicity of amyloid fibrils is described.

CrossMark
click for updates

OPEN ACCESS

Citation: Okoshi T, Yamaguchi I, Ozawa D, Hasegawa K, Naiki H (2015) Endocytosed 2-Microglobulin Amyloid Fibrils Induce Necrosis and Apoptosis of Rabbit Synovial Fibroblasts by Disrupting Endosomal/Lysosomal Membranes: A Novel Mechanism on the Cytotoxicity of Amyloid Fibrils. PLoS ONE 10(9): e0139330. doi:10.1371/journal.pone.0139330

Editor: Reza Khodarahmi, Kermanshah University of Medical Sciences, IRAN, ISLAMIC REPUBLIC OF

Received: March 27, 2015

Accepted: September 11, 2015

Published: September 30, 2015

Copyright: © 2015 Okoshi et al. This is an open access article distributed under the terms of the Creative Commons Attribution License, which permits unrestricted use, distribution, and reproduction in any medium, provided the original author and source are credited.

Data Availability Statement: All relevant data are within the paper and its Supporting Information files.

Funding: This work was supported in part by grants-in-aid for young scientists (B) 26860236 (TO) and scientific research (B) 25293094 (HN) from the Ministry of Education, Culture, Sports, Science, and Technology, Japan (http://www.mext.go.jp/), and for research on specific diseases "amyloidosis" H23-012 (HN) from the Ministry of Health, Labour and Welfare, Japan (http://www.mhlw.go.jp/). The funders had no

role in study design, data collection and analysis, decision to publish, or preparation of the manuscript.

Competing Interests: The authors have declared that no competing interests exist.

Introduction

Dialysis-related amyloidosis (DRA), is a systemic and nonhereditary amyloidosis that is a major and serious complication in long-term hemodialysis patients [1–3]. In DRA, β2-microglobulin (β2-m) amyloid fibrils deposit in the osteoarticular tissue, leading to carpal tunnel syndrome and destructive arthropathy with cystic bone lesions [4, 5]. Almost all amyloid fibrils deposited in the synovial membrane of the carpal tunnel consist of wild-type β2-m [6–9]. Although the increased concentration of β2-m in the plasma appears to be prerequisite [10], other factors, such as the age of the patient, the duration of hemodialysis, and less confidently the type of dialysis membrane used, may be involved [11–13]. A proinflammatory state induced by dialyzer membranes and contaminated dialysate may also contribute to the pathogenesis of DRA [14]. Histologically, this type of amyloidosis is characterized by marked infiltration of activated macrophages around the amyloid deposits [15–18]. These macrophages are considered to cause chronic destructive inflammation in the osteoarticular tissue [15, 16], and/or to play a role in the formation or degradation of β2-m amyloid fibrils [17, 18]. The molecular basis of the formation of β2-m amyloid fibrils has been explored intensively [19–21], but the mechanism by which the deposition of these amyloid fibrils causes the destruction of bone and joint tissue is not fully understood.

For various amyloidogenic proteins, different aggregation species such as mature fibrils, protofibrils, and oligomers, have been shown to possess individual cytotoxic potentials, and the mechanistic details of the cytotoxicity have been extensively investigated [22–24]. Although many investigators proposed that soluble oligomeric species of Alzheimer's amyloid-β (Aβ) protein and other amyloidogenic proteins may be the real culprit causing cytotoxicity and cellular dysfunction [24, 25], mature amyloid fibrils have also been shown to be cytotoxic under some conditions [26–30]. Various mechanisms have been proposed to explain the cytotoxicity and cellular dysfunction caused by these aggregation species. First, many investigators proposed the interaction of various aggregation species with plasma membranes [27, 29, 30–34], inducing direct disruption of membranes [31, 34], apoptosis via rise in cytosolic Ca^{2+} [32], and abnormal accumulation and overstabilization of raft domains in the membrane [33]. Other mechanisms include oxidative stress induced by catalase deactivation and rise in cytosolic H_2O_2 [35], Ca^{2+} release from the endoplasmic reticulum [36], neuroinflammation induced by microglia activation [28], and release of mitochondrial enzymes via the interaction with mitochondrial membranes [37].

Recently, Radford's group reported that β2-m amyloid fibrils are cytotoxic to many cell types [30, 38–40]. Xue et al [30] reported that β2-m amyloid fibrils disrupt membranes and reduce cell viability. Porter et al [38] reported that β2-m amyloid fibrils are cytotoxic to monocytes, impair the formation of bone resorbing osteoclasts from monocytes and reduce the viability of osteoblasts and chondrocytes. Very recently, Jakhria et al [40] reported that fragmented β2m amyloid fibrils accumulate in lysosomes of SH-SY5Y neuroblastoma cells, alter the trafficking of lysosomal membrane proteins, and inhibit the degradation of a model protein substrate by lysosomes. This study clearly showed that the dysfunction of lysosomes injured by amyloid fibrils may be responsible for the cytotoxicity of amyloid fibrils.

In this study, we assessed the effect of β2-m amyloid fibrils on the cultured synovial fibroblasts derived from rabbit periarticular soft tissue (HIG-82 cells) [41]. We found that endocytosed amyloid fibrils exhibit cytotoxicity by disrupting endosomal/lysosomal membranes, leading to both necrosis (plasma membrane disruption) and apoptosis. We propose a novel cytotoxic mechanism on the bone and joint disruption caused by β2-m amyloid fibrils.

Materials and Methods

Materials

Ham's F12 medium, penicillin-streptomycin mixed solution, Congo red, and SDS were obtained from Nacalai tesque Inc. (Kyoto, Japan). 1,2-Dimyristoyl-*sn*-glycero-3-phosphocholine (DMPC) and 1,2-dioleoyl-*sn*-glycero-3-phospho-(1'-*rac*-glycerol) (sodium salt) (DOPG) were obtained from Avanti Polar Lipids Inc. (Alabaster, AL, USA). PBS (-) solution for cell culture was obtained from Wako Pure Chemical Industries, Ltd. (Kanagawa, Japan). 5(6)-Carboxyfluorescein and cytochalasin D (CytoD) were obtained from Sigma (St. Louis, MO, USA).

Cell culture

HIG-82 rabbit synoviocyte cell line [41] was obtained directly from DS Pharma Biomedical Co., Ltd. (Osaka, Japan) (the catalogue number: 09–1832), and cultured in Ham's F12 medium supplemented with 10% fetal bovine serum (HyClone®, GE Healthcare Life Sciences, Logan, UT, USA), 100 units/ml penicillin and 100 μg/ml streptomycin at 37°C in an atmosphere of 5% CO_2.

Preparation of β2-m amyloid fibrils

Recombinant human β2-m (r-β2-m) was expressed and purified using the *Escherichia coli* expression system as described [42]. Protein concentration of r-β2-m was determined from the molar absorption coefficient (ε = 19 181 M^{-1} cm^{-1}) at 280 nm. β2-m amyloid fibrils used for all experiments in this study were prepared from the patient-derived β2-m amyloid fibrils by the repeated extension reaction at pH 7.5 with r-β2-m, as described elsewhere [43, 44]. Briefly, we obtained β2-m amyloid fibrils by incubating reaction mixture containing 30 μg/ml seeds, 25 μM r-β2-m, 50 mM phosphate buffer (pH 7.5), 100 mM NaCl, and 0.5 mM SDS for more than 24 hours at 37°C. To remove residual SDS and r-β2-m, the fibril solution was centrifuged at 15 000 rpm for 90 min, the supernatant exchanged for fresh PBS (-) solution, and extensively sonicated with 10 to 20 intermittent pulses (pulse = 0.6 s; interval = 0.4 s; output level = 2) using an ultrasonic disruptor UD-201 (TOMY SEIKO CO., LTD, Tokyo, Japan). This procedure was repeated four more times, and the final fibril solution was stored at 4°C until use. The stock solution contained fragmented fibrils with few monomers, but no soluble oligomeric species (S1 and S2 Figs). The protein concentration of β2-m amyloid fibrils was determined by the method using bicinchoninic acid [45] and a commercial protein assay kit (Micro BCA™ Protein Assay Kit, code 23235, Thermo Scientific, Rockford, IL, USA). The r-β2-m solution quantified as described above was used as the standard.

Light microscopy (LM) and electron microscopy (EM)

For LM observation, HIG-82 cells cultured on cover glasses in a 24-well plate, were incubated with Ham's F12 medium containing vehicle buffer (PBS (-)) or 100 μg/ml β2-m fibrils or r-β2-m monomer for 2 days, washed with PBS (-) solution, then fixed with 100% ethanol and stained with CnT-S-100 Stain Kit (CELLnTEC, Bern, Switzerland) according to the manufacturer's instructions. The stained and dried cover glasses were mounted on glass slides and the images were taken with a light microscope (BX51, Olympus, Tokyo, Japan) equipped with a CCD camera (DP26, Olympus). For EM observation, HIG-82 cells cultured in 35 mm dishes were incubated with Ham's F12 medium containing vehicle buffer or 100 μg/ml β2-m fibrils or r-β2-m monomer for 2 to 6 hrs, prefixed with 2% paraformaldehyde and 2% glutaraldehyde in 30 mM HEPES, 100 mM NaCl, and 2 mM $CaCl_2$ (pH 7.4), and postfixed with 1% osmium tetroxide in the same buffer at 4°C for 30 min. After staining with 3% uranyl acetate for 30 to

60 min and embedding in epoxy resin (Quetol 812, Nissin EM, Tokyo, Japan), the ultrathin sections of the cells were made and double-stained with uranyl acetate and lead citrate. The images were digitally taken with Hitachi H-7650 transmission electron microscope with an acceleration voltage of 80 kV.

LDH release assay and MTT reduction assay

For lactate dehydrogenase (LDH) release assay, HIG-82 cells were incubated in a 24-well plate with Ham's F12 medium containing vehicle buffer (PBS (-)), 10 or 100 μg/ml β2-m fibrils or r-β2-m monomer for 2 days. Medium was collected from each well, centrifuged at 4 000 rpm for 5 min to precipitate floating cells, and the activity of LDH released from damaged cells into the medium was measured with a Cytotoxicity Detection Kit^{PLUS} (Roche, Mannheim, Germany) according to the manufacturer's instructions. For 3-(4,5-di-methylthiazol-2-yl)-2,5-diphenyl-tetrazolium bromide (MTT) reduction assay, after incubating with β2-m fibrils or r-β2-m monomer for 2 days, the MTT reduction activity of adherent HIG-82 cells was measured with Cell Proliferation Kit I (Roche) according to the manufacturer's instructions. In both assays, the absorbance was measured with a 96-well plate and a microplate reader (SpectraMax 250 Microplate Reader, Molecular Devices, Sunnyvale, CA, USA). To analyze the effect of CytoD, HIG-82 cells were preincubated in a 24-well plate with Ham's F12 medium containing 0 to 1.0 μg/ml CytoD for 2 hrs prior to the addition of vehicle buffer or 100 μg/ml β2-m fibrils. After incubating with vehicle or fibrils in the presence of CytoD for 2 days, LDH release assay and MTT reduction assay were performed as described above. Data were presented as mean ± SD of three independent experiments.

TUNEL assay

After HIG-82 cells were incubated in a 24-well plate with Ham's F12 medium containing vehicle buffer or 100 μg/ml β2-m fibrils or r-β2-m monomer for 2 days, they were washed with PBS (-) two times. Then, the terminal deoxynucleotidyl transferase-mediated biotinylated UTP nick end labeling (TUNEL) assay was performed for adherent cells with In Situ Cell Death Detection Kit, TMR red (Roche) according to the manufacturer's instructions. As a positive control, cells were incubated with 1 000 unit/ml DNase I recombinant, RNase-free (Roche) for 10 min at room temperature to break DNA strand, prior to labeling procedure. After TUNEL reaction, nuclei were counterstained with DAPI (DAPI solution, PromoCell, Heidelberg, Germany or DAPI Nucleic Acid stain, Lonza Walkersville, Inc., Walkersville, MD, USA) according to the manufacturer's instructions. Samples were analyzed under a fluorescence microscope (IX70, Olympus) equipped with a CCD camera (DP70, Olympus) using a 520–550 nm or 330–385 nm excitation filter, and a 580~ nm or 420~ nm band pass filter for TUNEL and DAPI assays, respectively. Images of 4 microscopic fields at 100-fold magnification were captured randomly, and the number of TUNEL-positive cells and DAPI-positive nuclei was counted manually to calculate the percentage of apoptotic cells to total cells. Data were presented as a dot plot of the percentage of five independent experiments with the mean value.

Fluorescent dye release assay from liposome

Large unilamellar vesicles (LUV) containing carboxyfluorescein were prepared as described elsewhere [30] with minor modifications. Briefly, mixed lipid powder (6.2 mg DMPC and 1.6 mg DOPG) was dissolved in a small amount of chloroform in a round bottom flask, and the solvent was evaporated in a fume hood overnight. The obtained lipid film was hydrated with 4 ml PBS containing 50 mM carboxyfluorescein. The lipid suspension was put through 5 freeze-thaw cycles before being extruded through 1.0 μm polycarbonate filters with a Mini-Extruder

apparatus (Avanti) according to the manufacturer's instructions. Then the LUVs were washed three times by centrifugation at 4 000 rpm for 5 min and resuspension in the same volume of PBS to remove free carboxyfluorescein. For a dye release assay, the resulting LUV solution was diluted 1 000-fold into PBS, then β2-m fibrils or r-β2-m monomer (final 0 or 100 μg/ml), or Triton X-100 as a positive control (final 2%) was added to the diluted solution. After incubation for up to 1 day, the fluorescence was measured with excitation/emission wavelengths of 492/517 nm using a Hitachi F-4500 fluorescence spectrophotometer.

Congo red staining

HIG-82 cells were stained with Congo red as described elsewhere [26, 46] with minor modifications. Briefly, HIG-82 cells cultured on a glass bottom culture dish (P35G-0-14-C, MatTek, Ashland, MA, USA), were incubated with Ham's F12 medium containing vehicle buffer or 100 μg/ml β2-m fibrils for 6 hrs, washed twice with Hanks' balanced salt solution, stained with 20 μM Congo red for 30 min, and washed twice with Hanks' balanced salt solution. The cells were observed using a Leica DMIRE2 inverted microscope equipped with Leica TCS SP2 AOBS spectral confocal scanning system (excitation, 543 nm; emission, 555–700 nm).

Lysotracker staining and indirect immunofluorescence for β2-m

HIG-82 cells cultured on a glass bottom culture dish (P35G-0-14-C, MatTek), were incubated with Ham's F12 medium containing vehicle buffer, 10 μg/ml β2-m monomer, or 10 μg/ml β2-m fibrils for 12 hrs, washed twice with culture medium, stained with lysotracker (Cell Navigator Lysosome Staining Kit, AAT Bioquest, Inc., Sunnyvale, CA, USA) according to the manufacturer's instructions, washed twice with culture medium, and fixed with 4% paraformaldehyde in PBS for 30 min at 37°C in the dark. Next, cells were washed three times with PBS (-), permeabilized with 0.2% Triton X-100 in PBS for 20 min at room temperature, blocked with 3% bovine serum albumin (BSA) (Nacalai tesque) in PBS for 1 hr at room temperature, incubated overnight with rabbit polyclonal anti-human β2-m antibodies (DAKO Japan, Tokyo, Japan) diluted 1:5 000 in PBS with 1% BSA at 4°C, and incubated for 1 hr with Alexa Fluor 488-conjugated goat anti-rabbit IgG polyclonal secondary antibodies (Life technologies Japan, Tokyo, Japan) diluted 1:5 000 in PBS with 1% BSA at room temperature. The stained cells were mounted with DAPI-containing mountant (SlowFade Diamond Antifade Mountatnt with DAPI, Life technologies) and observed using a Leica DMIRE2 inverted microscope equipped with Leica TCS SP2 AOBS spectral confocal scanning system (excitation, 405/ 488/ 543 nm; emission, 420-470/ 500-540/ 580–650 nm for DAPI/ Alexa Fluor 488-conjugated secondary antibody/ lysotracker, respectively).

Statistical analysis

Statistical analysis was performed by Student's unpaired t-test, except for TUNEL assay, for which Mann-Whitney U-test was carried out. A value of $p<0.05$ was considered statistically significant.

Results

Evaluation of the morphological changes by light microscopy

To assess the effect of β2-m amyloid fibrils on the proliferation and morphology of HIG-82 cells, we first observed HIG-82 cells by phase contrast microscopy after incubation with Ham's F12 medium containing vehicle buffer, 100 μg/ml r-β2-m monomer, and 10 or 100 μg/ml β2-m amyloid fibrils for up to 2 days. Only when HIG-82 cells were incubated with 100 μg/ml

amyloid fibrils for 2 days, many injured cells with round shape and floating in the medium were observed (S3 Fig). Then, we evaluated detailed morphological changes by light microscopy as described in Materials and Methods. When HIG-82 cells were incubated with Ham's F12 medium containing 100 μg/ml β2-m monomer or vehicle for 2 days, confluent proliferation of uniform spindle cells was observed in a fascicular or storiform pattern (Fig 1A and 1B). In contrast, when incubated with Ham's F12 medium containing 100 μg/ml amyloid fibrils for 2 days, sparse proliferation of injured cells with picnotic nuclei, swelling and vacuolation of the cytoplasm suggesting necrosis [47, 48], was observed (Fig 1C and 1D). Fragmentation of nuclei suggesting apoptosis [47, 48] was also observed (Fig 1C and 1D).

β2-m amyloid fibrils reduce cellular viability

We next quantified the cytotoxic effect of β2-m amyloid fibrils on HIG-82 cells by LDH releasing assay and MTT reduction assay as described in Materials and Methods. In LDH releasing assay (Fig 2A), 100 μg/ml β2-m amyloid fibrils reduced cellular viability significantly as compared to β2-m monomer and vehicle buffer (54.2 ± 5.1% of positive control vs. 11.2 ± 0.6% and 11.7 ± 0.5%, respectively; $P<0.0001$ in both cases). 10 μg/ml β2-m amyloid fibrils exhibited no significant cytotoxicity. These data suggest that β2-m amyloid fibrils induced the necrosis of HIG-82 cells, leading to the rupture of plasma membranes [47, 48]. In the MTT reduction assay (Fig 2B), 100 μg/ml β2-m amyloid fibrils reduced cellular viability significantly as compared to β2-m monomer and vehicle (25.3 ± 0.3% of vehicle vs. 93.3 ± 5.5% and 100%, respectively; $P<0.0001$ in both cases). 10 μg/ml β2-m amyloid fibrils also exhibited significant cytotoxicity as compared to vehicle (77.0 ± 12.5% of vehicle; $P<0.05$). The supernatant of the fibril preparation added to the cells did not affect cellular viability of HIG-82 cells as measured by the LDH releasing assay and the MTT reduction assay (S4 Fig), indicating that residual SDS contamination is not responsible for the observed cytotoxicity.

β2-m amyloid fibrils induce apoptosis

To further characterize the contribution of apoptosis to the cytotoxicity of β2-m amyloid fibrils, we performed the TUNEL assay as described in Materials and Methods (Fig 3). When HIG-82 cells were incubated with 100 μg/ml amyloid fibrils for 2 days, the percentage of apoptotic cells increased significantly as compared to β2-m monomer and vehicle buffer (2.9% of total cells vs. 0.3% and 0.3%, respectively; $P<0.05$ in both cases) (Fig 3B).

β2-m amyloid fibrils adhere to the cell surfaces, but don't destruct artificial plasma membranes

To further characterize the effect of β2-m amyloid fibrils on HIG-82 cells, we performed Congo red staining and investigated whether β2-m amyloid fibrils directly interacted with the plasma membranes of HIG-82 cells. When HIG-82 cells were incubated with 100 μg/ml amyloid fibrils for 6 hrs, they were firmly covered with amyloid fibrils (Fig 4). This observation may indicate that β2-m amyloid fibrils adhered to the cell surface may directly injure the plasma membrane, leading to the release of LDH (Fig 2A). Thus, we next investigated the ability of β2-m amyloid fibrils to destruct artificial plasma membranes by the liposome dye release assay (Fig 5). As shown in Fig 5B and 5C, β2-m amyloid fibrils did not significantly destruct artificial plasma membranes of LUVs.

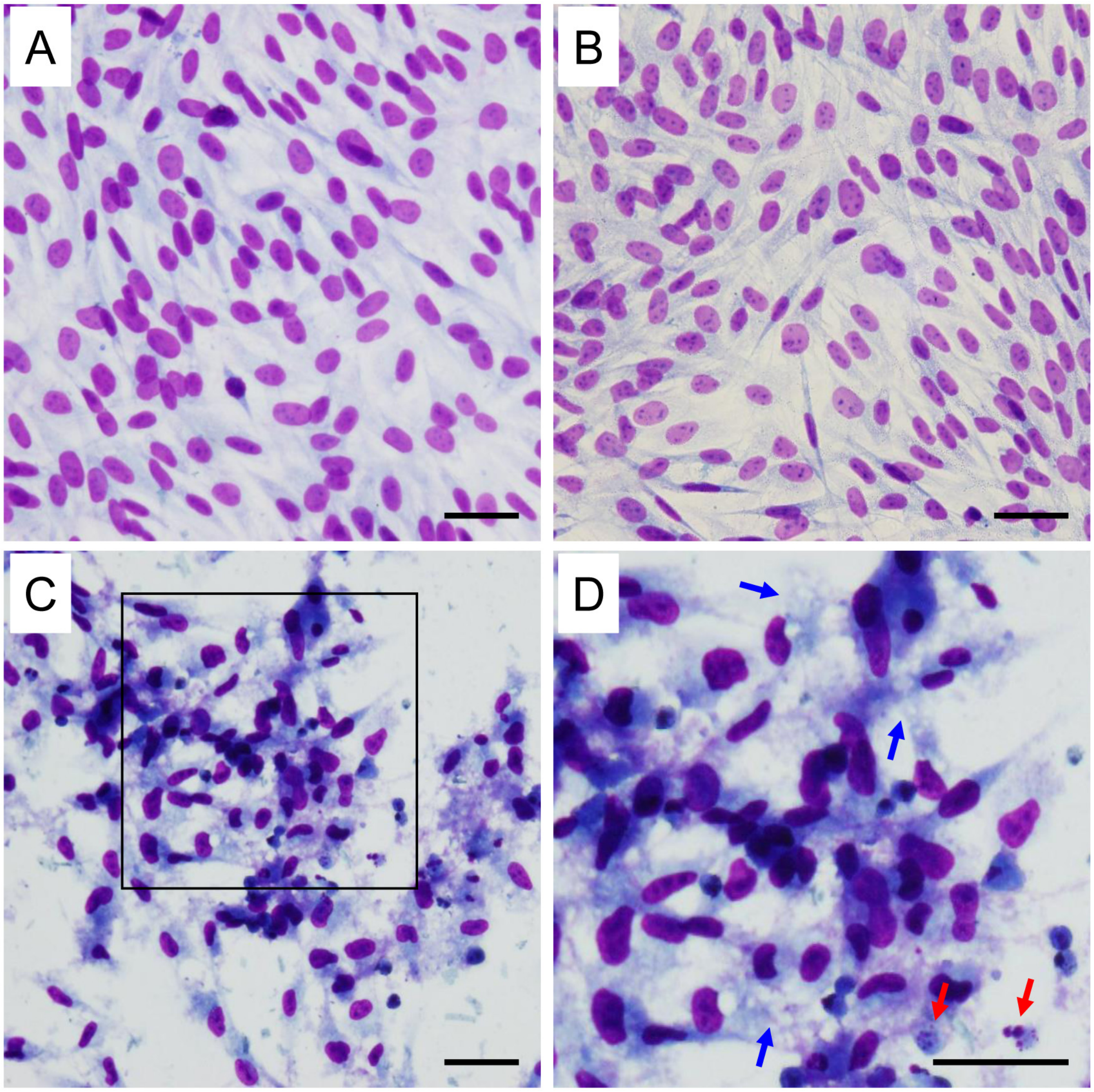

Fig 1. HIG-82 cells incubated with β2-m amyloid fibrils exhibit both necrotic and apoptotic changes morphologically. Representative light micrographs of HIG-82 cells incubated with Ham's F12 medium containing vehicle buffer (PBS (-)) (A), 100 μg/ml r-β2-m monomer (B), or 100 μg/ml β2-m amyloid fibrils (C) for 2 days as described in Materials and Methods. (D) A higher magnification of the box in (C). (A, B) Confluent proliferation of uniform spindle cells was observed in a fascicular or storiform pattern. (C, D) In contrast, sparse proliferation of injured cells with picnotic nuclei, swelling and vacuolation of the cytoplasm suggesting necrosis, was observed. Fragmentation of nuclei suggesting apoptosis was also observed. In (D), blue and red arrows indicate swelling and vacuolation of the cytoplasm and fragmentation of nuclei, respectively. The scale bars are 50 μm long.

doi:10.1371/journal.pone.0139330.g001

β2-m amyloid fibrils are internalized and sorted to lysosomes

To examine the possibility that β2-m amyloid fibrils are endocytosed and sorted to lysosomes by HIG-82 cells, we next performed lysotracker staining and indirect immunofluorescence for β2-m, followed by confocal laser microscopy as described in Materials and Methods (Fig 6). When the cells were incubated with fibrils (Fig 6, right column), green fluorescence indicating β2-m fibrils were observed inside the cells in a granular pattern, as well as on the surface of the cells. Interestingly, some green-colored granules containing β2-m fibrils were merged with red-colored lysosomes, indicating that β2-m fibrils were carried by the endosomal-lysosomal pathway.

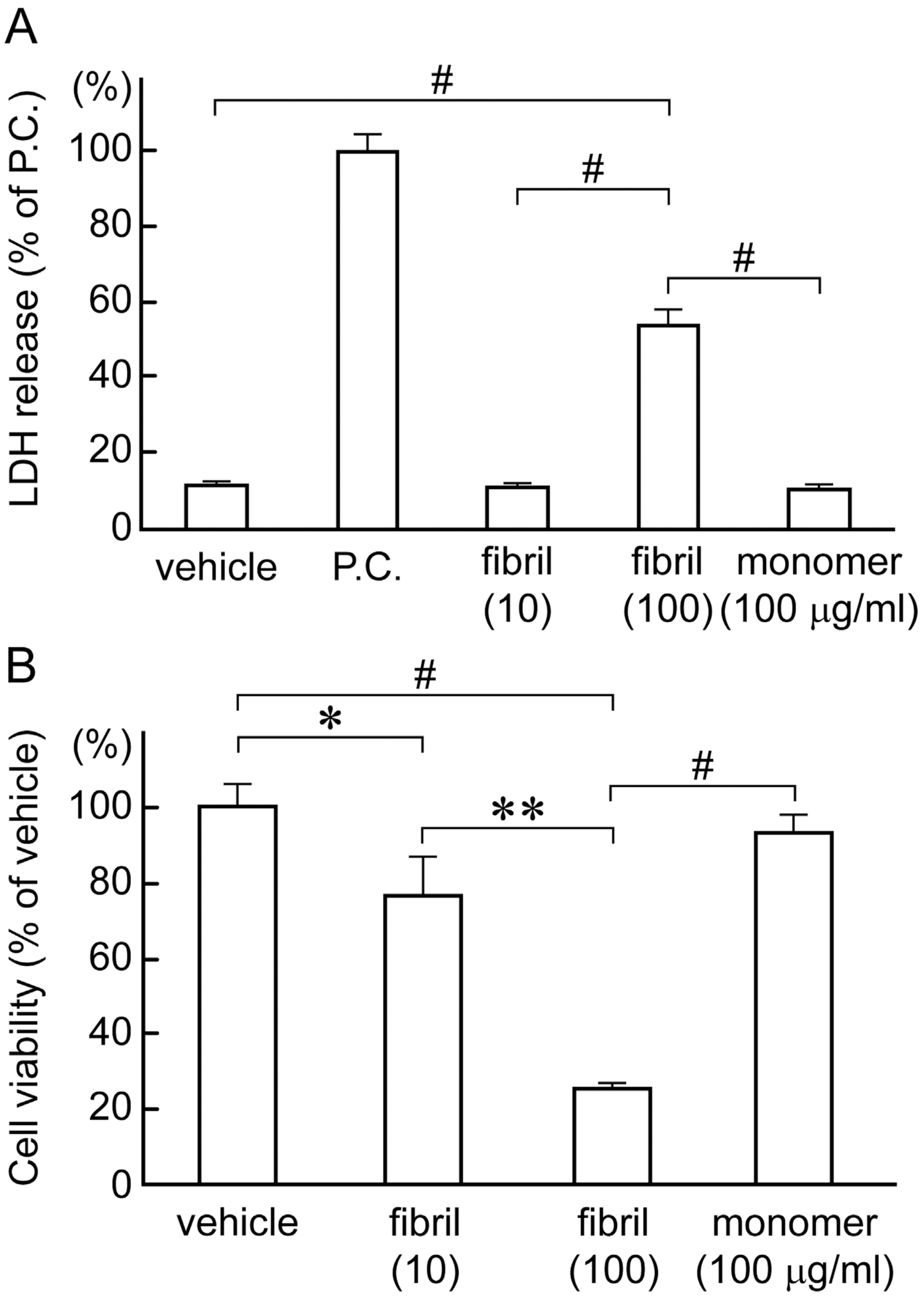

Fig 2. β2-m amyloid fibrils reduce cellular viability of HIG-82 cells. After HIG-82 cells were incubated with Ham's F12 medium containing vehicle buffer, 10 or 100 μg/ml β2-m fibrils or r-β2-m monomer for 2 days, LDH releasing assay (A) and MTT reduction assay (B) were performed as described in Materials and Methods. Data normalized to positive control and vehicle in LDH releasing assay and MTT reduction assay, respectively were presented as mean ± SD of three independent experiments. Statistical analysis was performed by Student's unpaired t-test. *P < 0.05, **P < 0.001, $^{\#}$P < 0.0001.

doi:10.1371/journal.pone.0139330.g002

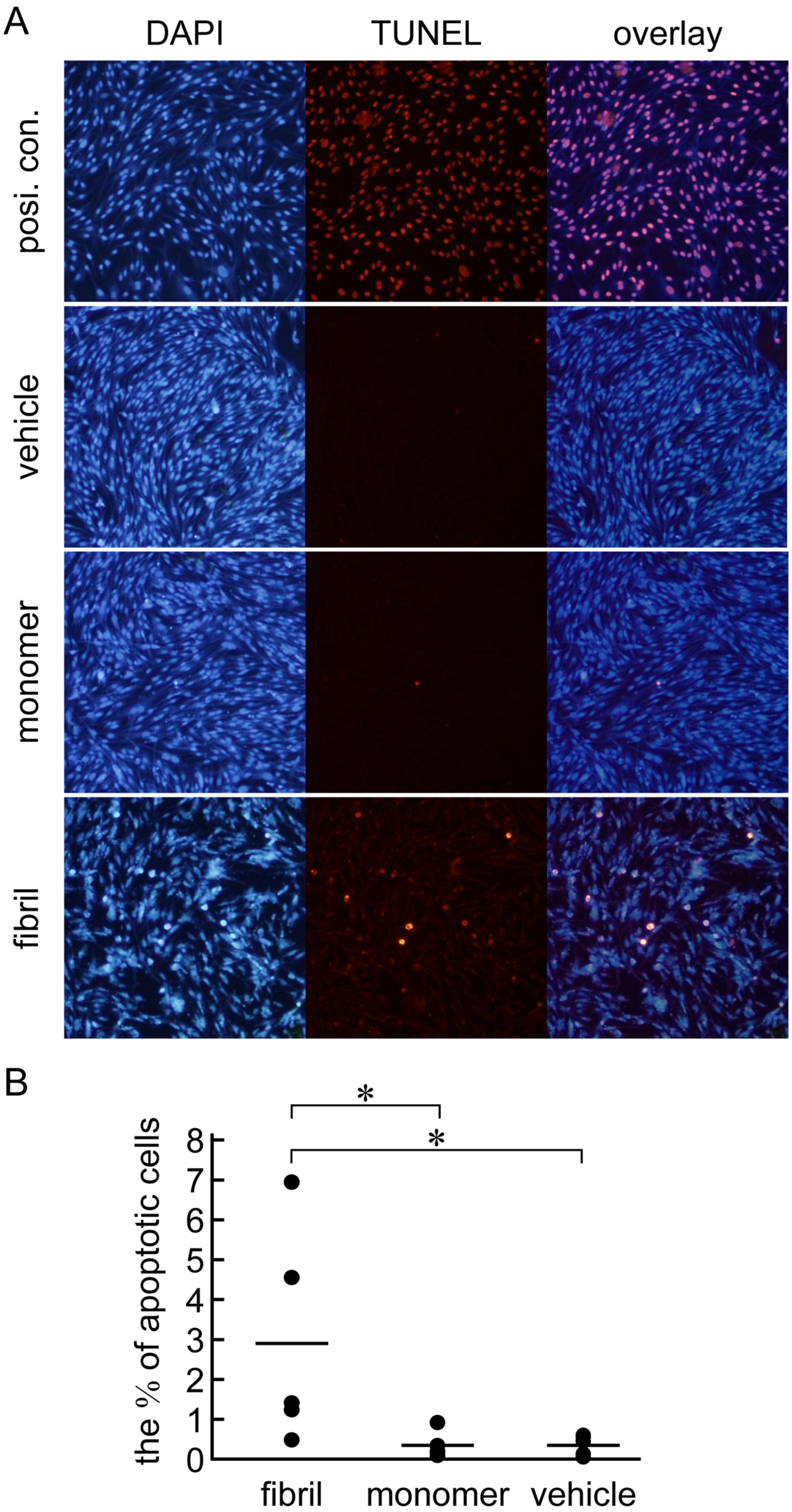

Fig 3. β2-m amyloid fibrils induce apoptosis of HIG-82 cells as measured by the TUNEL assay. After HIG-82 cells were incubated with Ham's F12 medium containing vehicle buffer or 100 μg/ml β2-m fibrils or r-β2-m monomer for 2 days, TUNEL assay was performed as described in Materials and Methods. (A) The representative fluorescence images of TUNEL and DAPI double staining. The original magnification was

x100. (B) The percentage of apoptotic cells to total cells. Data were presented as a dot plot of the ratios of five independent experiments with the mean value. Statistical analysis was performed by Mann-Whitney U-test. *P < 0.05.

doi:10.1371/journal.pone.0139330.g003

Evaluation of the morphological changes by transmission electron microscopy

To investigate the mechanism of cytotoxicity mediated by amyloid fibrils, we next performed the ultrastructural analysis with transmission electron microscopy. When HIG-82 cells were incubated with Ham's F12 medium containing vehicle buffer for 6 hrs, the cellular outline was clear and smooth. The oval nucleus with thin nuclear membrane, fine and homogenous chromatin, and a few nucleoli was observed (Fig 7A). In contrast, when HIG-82 cells were incubated with Ham's F12 medium containing 100 μg/ml amyloid fibrils for 2 hrs, they were covered with amyloid fibrils (Fig 7B). Moreover, a part of the plasma membrane covered with amyloid fibrils was found to invaginate and fuse to form an endocytic vesicle containing amyloid fibrils (Fig 7B, inset). There were many intracytoplasmic endosomes/lysosomes filled with amyloid fibrils (Fig 7B) and some endosomal/lysosomal membranes were disrupted by intravesicular fibrils (Fig 7C). When HIG-82 cells were incubated with amyloid fibrils for 6 hrs, the cytoplasm was filled with endosomes/lysosomes containing abundant amyloid fibrils and some endosomes/lysosomes were found to fuse with each other (Fig 7E). Very importantly, the endocytosed amyloid fibrils leaked from endosomal/lysosomal vesicles into the cytosol and were

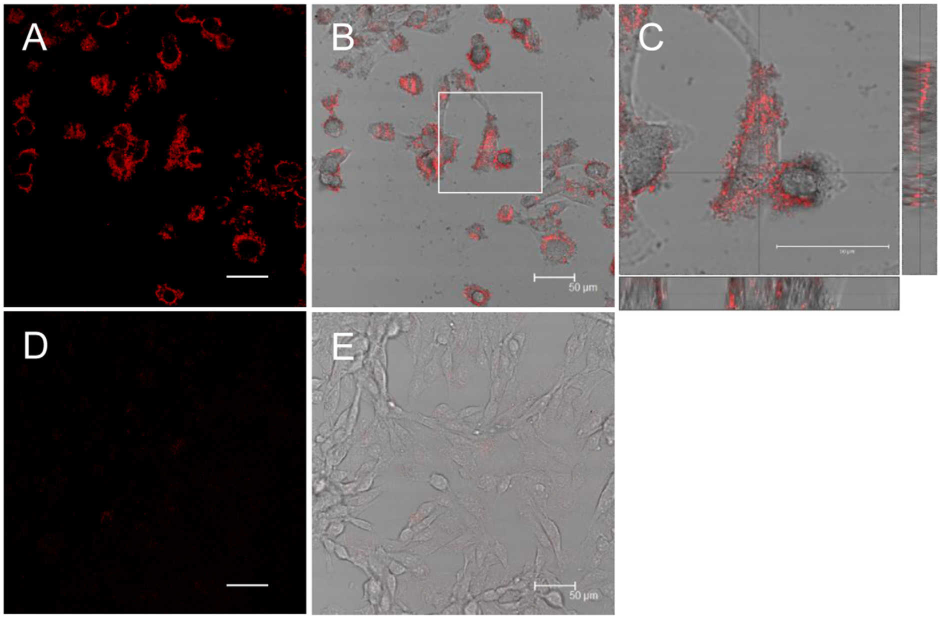

Fig 4. β2-m amyloid fibrils adhere to the cell surfaces. HIG-82 cells incubated with Ham's F12 medium containing 100 μg/ml β2-m fibrils (A-C) or vehicle buffer (D, E) for 6 hrs were stained with Congo red and observed with the confocal laser microscope as described in Materials and Methods. (B) and (E) are representative superimposed images on individual bright field micrographs. (C) A higher magnification of the box in (B). Images attached on the right and bottom are those of vertical sections on the lines intersecting at right angles. (A-C) When HIG-82 cells were incubated with 100 μg/ml amyloid fibrils for 6 hrs, they were firmly covered with amyloid fibrils. The scale bars are 50 μm long.

doi:10.1371/journal.pone.0139330.g004

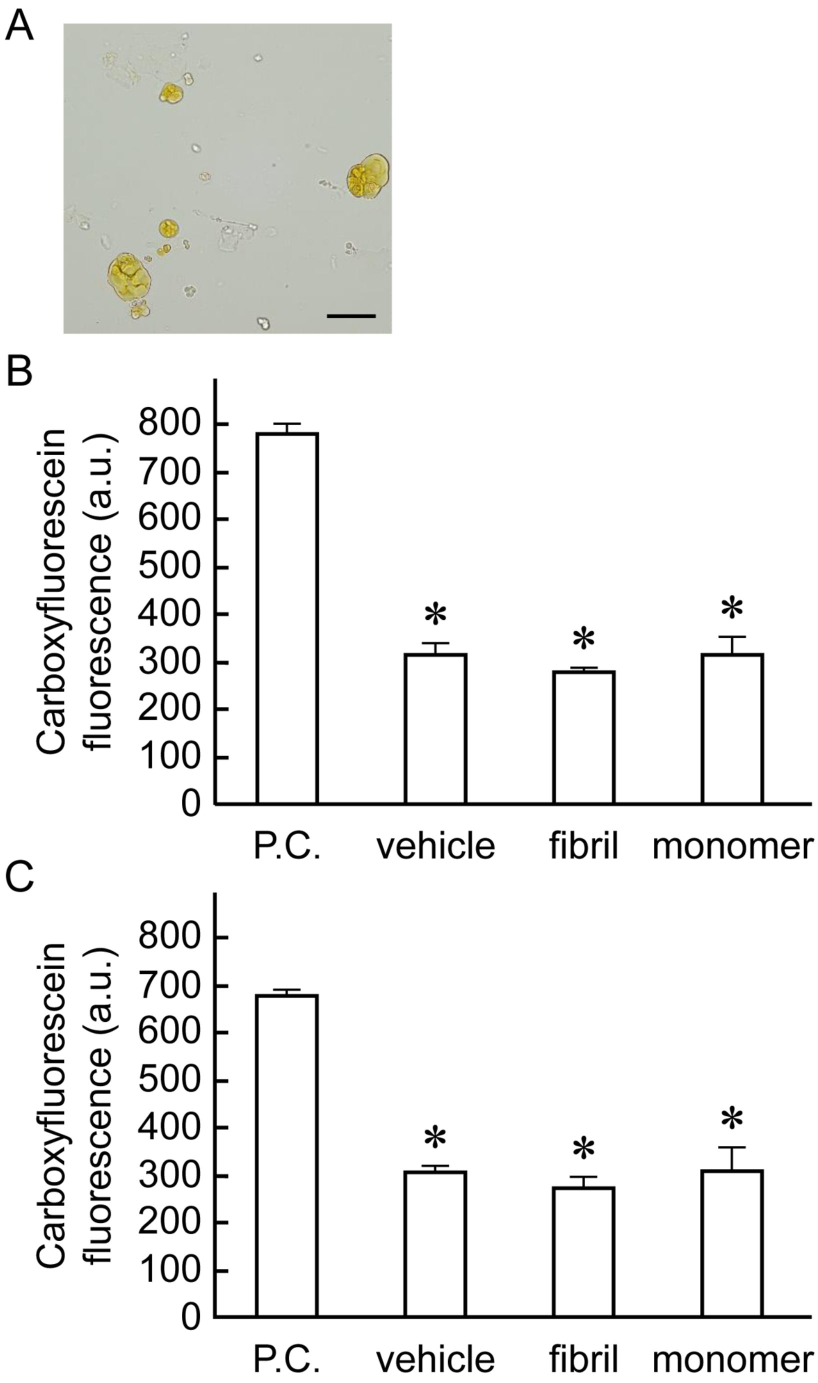

Fig 5. β2-m amyloid fibrils have no effect on artificial plasma membranes. (A) A representative light micrograph of large unilamellar vesicles (LUVs) containing carboxyfluorescein prepared as described in Materials and Methods. They were less than 50 μm in diameter. The scale bars are 50 μm long. After LUVs were incubated with β2-m fibrils or r-β2-m monomer (final 0 or 100 μg/ml), or Triton X-100 as a positive control (final 2%) for 15 min (B) or 1 day (C), the fluorescence was measured as described in Materials and Methods. (B, C) β2-m amyloid fibrils did not significantly destruct artificial plasma membranes of LUVs. Statistical analysis was performed by Student's unpaired t-test. *$P < 0.05$ vs. positive control.

doi:10.1371/journal.pone.0139330.g005

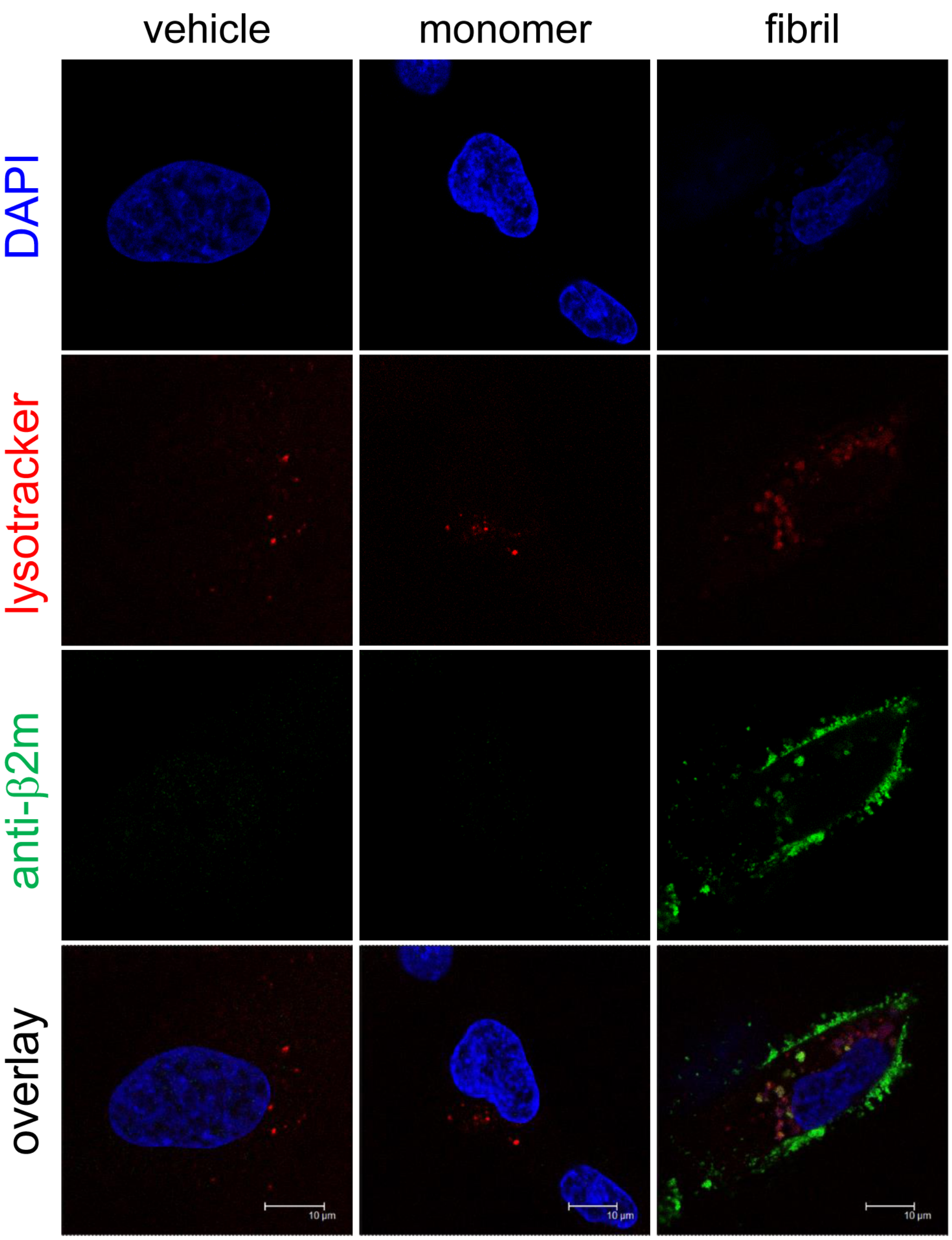

Fig 6. β2-m amyloid fibrils are internalized and sorted to lysosomes. HIG-82 cells incubated with Ham's F12 medium containing vehicle buffer, 10 μg/ml β2-m monomer, or 10 μg/ml β2-m fibrils for 12 hrs were stained for lysosomes (red), β2-m (green), and nuclei (blue), and observed with the confocal laser microscope as described in Materials and Methods. When the cells were incubated with fibrils (right column), green fluorescence indicating β2-m fibrils were observed inside the cells in a granular pattern, as well as on the surface of the cells. Importantly, some green-colored granules containing β2-m fibrils were merged with red-colored lysosomes. The scale bars are 10 μm long.

doi:10.1371/journal.pone.0139330.g006

contiguous with actin filaments (Fig 8A and 8D), and some amyloid fibrils were found adjacent to mitochondria (Fig 8B, 8C, 8E, and 8F). Nuclear deformation, shrinkage, and chromatin condensation at the nuclear rim (Fig 7F) and partial disruption of plasma membranes were also observed (Fig 7D).

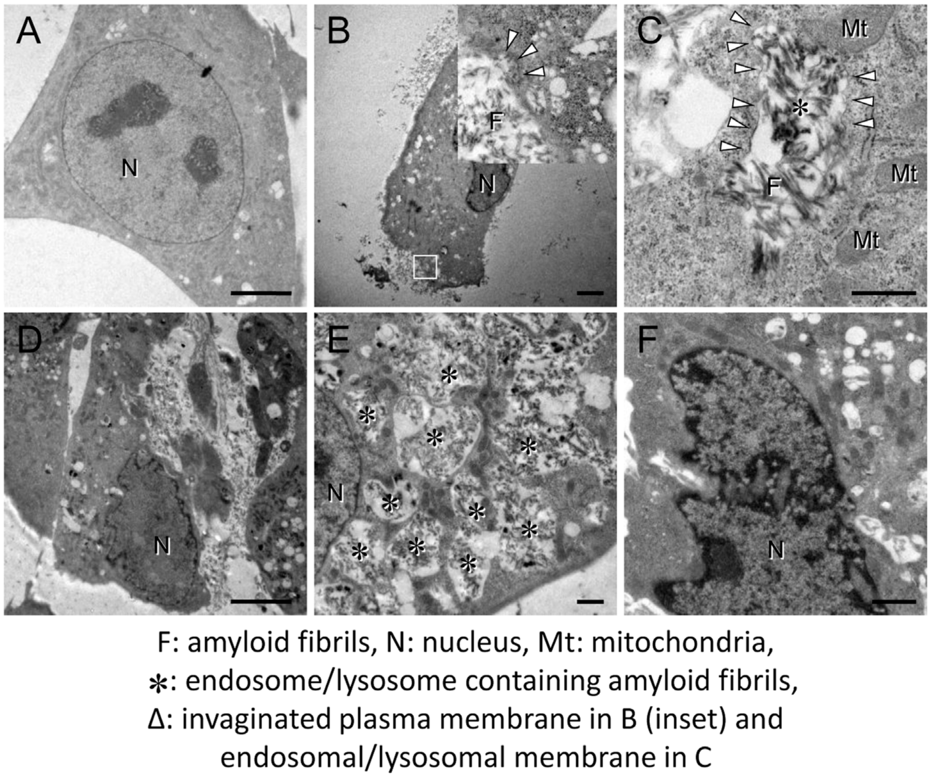

Fig 7. β2-m amyloid fibrils are endocytosed into endosomes/lysosomes, leading to the disruption of their membranes. Representative electron micrographs of HIG-82 cells taken as described in Materials and Methods. HIG-82 cells were incubated with Ham's F12 medium containing vehicle buffer for 6 hrs (A), or 100 μg/ml β2-m fibrils for 2 hrs (B, C) or 6 hrs (D-F) as described in Materials and Methods. The inset in (B) is a higher magnification of the box. (B, D) HIG-82 cells were covered with amyloid fibrils. Note that a part of the plasma membrane invaginated and fused to form an endocytic vesicle containing amyloid fibrils (inset in B). (C, E) Many endosomes/lysosomes were filled with amyloid fibrils, and some endosomal/lysosomal membranes were disrupted by intravesicular fibrils. (F) Nuclear deformation, shrinkage, and chromatin condensation at the nuclear rim were also observed. The scale bars are 5 μm long in A, B, D and F and 1 μm long in C and E.

doi:10.1371/journal.pone.0139330.g007

Inhibition of endocytosis attenuates the toxicity of β2-m amyloid fibrils

To further confirm the contribution of endosomes/lysosomes to the cytotoxicity of β2-m amyloid fibrils, we finally examined the effect of CytoD on the cytotoxicity of β2-m amyloid fibrils. CytoD, a kind of fungal toxin from *Zygosporium mansonii*, disrupts actin polymerization and inhibits actin-dependent endocytosis at 1.0 μg/ml [49, 50]. As shown in Fig 9, CytoD dose-dependently attenuated the cytotoxicity of β2-m amyloid fibrils in both the LDH releasing assay (33.1 ± 3.4%, 33.2 ± 5.2%, 24.4 ± 2.4% and 20.0 ± 1.3% of positive control for 0, 0.1, 0.5 and 1.0 μg/ml CytoD, respectively; 0 vs. 1.0 μg/ml CytoD, $P<0.01$; 0 vs. 0.5 and 0.1 vs. 1.0 μg/ml CytoD, $P<0.05$) and the MTT reduction assay (59.7 ± 9.9%, 58.6. ± 10.2%, 74.8 ± 6.8% and 78.1 ± 5.0% of the negative control for 0, 0.1, 0.5 and 1.0 μg/ml CytoD, respectively; 0 vs. 1.0 and 0.1 vs. 1.0 μg/ml CytoD, $P<0.05$).

Discussion

In the present study, we assessed the cytotoxic effect of β2-m amyloid fibrils on the cultured rabbit synovial fibroblasts (HIG-82 cells). Since HIG-82 is a continuous cell line from soft

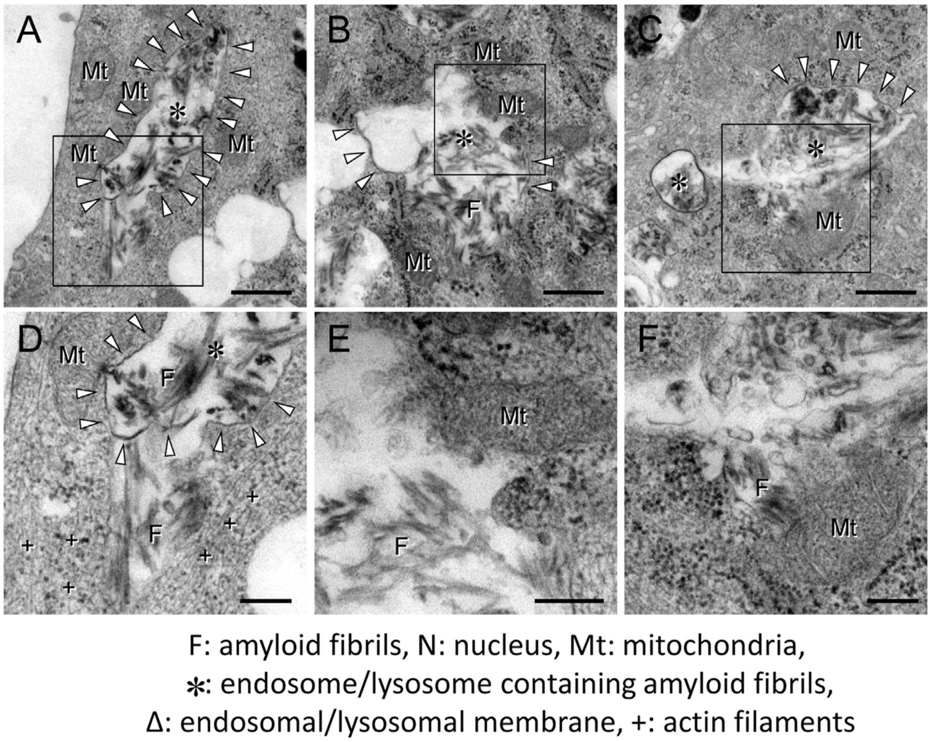

Fig 8. Endocytosed β2-m amyloid fibrils leak from endosomes/lysosomes into the cytosol. Representative electron micrographs of HIG-82 cells incubated with Ham's F12 medium containing 100 μg/ml β2-m fibrils for 6 hrs as described in Materials and Methods. Images were taken as described in Materials and Methods. (D-F) Higher magnifications of the boxes in A-C, respectively. Note that the endocytosed amyloid fibrils leaked from endosomal/lysosomal vesicles into the cytosol (A, D), and some fibrils were found adjacent to mitochondria (B, C, E, F). The scale bars are 500 nm long in A-C and 200 nm long in D-F.

doi:10.1371/journal.pone.0139330.g008

tissue lining the knee joints of rabbits and considered to be suitable for the research into the pathophysiology of various arthritides [41], it may be reasonable to use this cell line for investigating the mechanism by which the deposition of β2-m amyloid fibrils in DRA causes the destruction of bone and joint tissue. We first demonstrated that β2-m amyloid fibrils had a cytotoxic effect on HIG-82 cells, leading to both necrotic and apoptotic changes of these cells (Figs 1–3). We next investigated the mechanisms of cytotoxicity caused by β2-m amyloid fibrils and found that many intracytoplasmic endosomes/lysosomes were filled with amyloid fibrils, and some endosomal/lysosomal membranes were disrupted by intravesicular fibrils (Figs 6–8). Interestingly, some endocytosed amyloid fibrils leaked from endosomal/lysosomal vesicles into the cytosol and were adjacent to mitochondria (Fig 8). These results suggest that endocytosed β2-m amyloid fibrils could induce necrosis and apoptosis by directly disrupting endosomal/lysosomal membranes.

Many research groups indicated that the endosome/lysosome system is deeply involved in the cytotoxicity caused by various species of misfolded and aggregated proteins. Jakhria et al [40] reported that fragmented β2-m amyloid fibrils disrupt lysosomal membrane protein trafficking and inhibit protein degradation by lysosomes. They suggested that nanosized fibrils formed early during amyloid assembly reactions or by the fragmentation of longer fibrils could play a role in amyloid disease by deteriorating the function of the endosomal/lysosomal pathway. Although they did not observe any cell death by incubation of cells with fragmented β2-m fibrils, this may be mainly due to the difference in the cell line used in their experiments

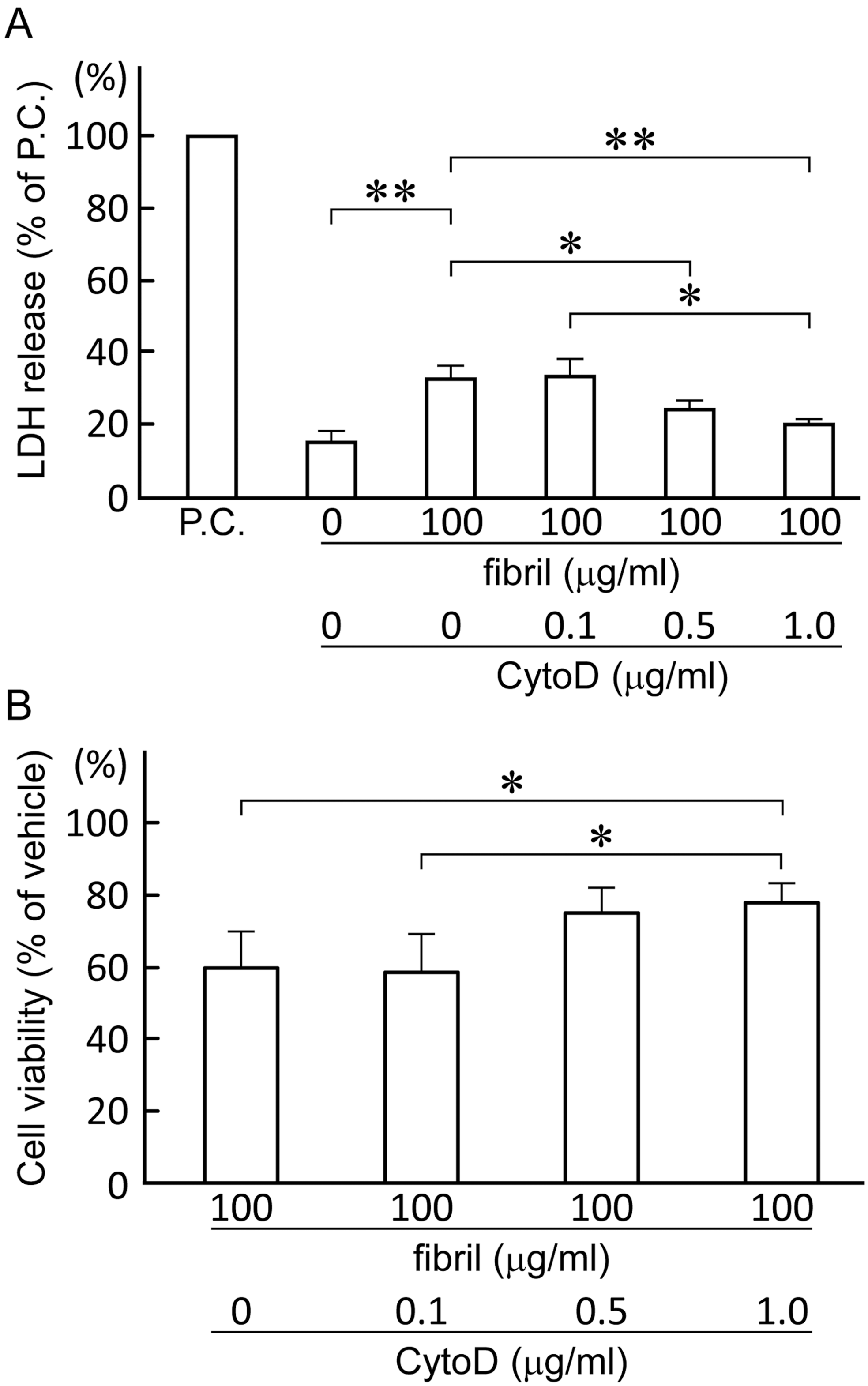

Fig 9. Inhibition of endocytosis by cytochalasin D attenuates the toxicity of β2-m amyloid fibrils. HIG-82 cells preincubated with Ham's F12 medium containing 0 to 1.0 μg/ml cytochalasin D (CytoD) for 2 hrs, were incubated with the medium containing vehicle buffer or 100 μg/ml β2-m fibrils in the presence of CytoD for 2 days. LDH releasing assay (A) and MTT reduction assay (B) were performed as described in Materials

and Methods. Data normalized to positive control and vehicle in LDH releasing assay and MTT reduction assay, respectively, were presented as mean ± SD of three independent experiments. Statistical analysis was performed by Student's unpaired t-test. *P < 0.05, **P < 0.01.

doi:10.1371/journal.pone.0139330.g009

(SH-SY5Y neuroblastoma cells vs. HIG-82 cells). Ditaranto et al [51] demonstrated that soluble Aβ1–42 internalized from the culture medium accumulates inside the endosomes/lysosomes, invoking the free radical generation within lysosomes and disruption of lysosomal membrane proton gradient, thus leading to cell death. Umeda et al [52] reported that in transgenic mice expressing E693Δ mutation of amyloid precursor protein, Aβ oligomer accumulated in the endosomes/lysosomes causes cell death by inducing leakage of cathepsin D from endosomes/lysosomes into cytoplasm. Guan et al [53] reported that impaired lysosomal function is the major cause of amyloidogenic light chain-induced proteotoxicity. However, to the best of our knowledge, there is no report indicating that endocytosed amyloid fibrils directly disrupt endosomal/lysosomal membranes. Therefore, our current observations may propose the novel and attractive cytotoxic mechanism of amyloid fibrils, that is, the disruption of endosomal/lysosomal membranes by endocytosed amyloid fibrils followed by the leakage of lysosomal enzymes, as well as by the interaction of amyloid fibrils with cytoplasmic proteins [35] and mitochondrial membranes [37].

HIG-82 cells have phagocytic properties like synoviocytes [41, 54, 55] and CytoD attenuated the cytotoxicity of β2-m amyloid fibrils for HIG-82 cells (Fig 9). CytoD inhibits phagocytosis by both mononulcear phagocytes and polymorphonuclear leukocytes [49], by inhibiting elongation of the actin filament at both ends [50]. However, CytoD does not affect pinocytosis of mononuclear phagocytes and other eukaryotic cells [49]. Interestingly, Jakhria et al [40] indicated that dynamin-dependent endocytosis of fragmented β2-m amyloid fibrils is required for their cytotoxicity for the SH-SY5Y neuroblastoma cell line. Since CytoD did not inhibit the toxicity of β2-m amyloid fibrils completely (Fig 9) and the intracytoplasmic vesicles containing amyloid fibrils were observed in HIG-82 cells preincubated with CytoD (S5 Fig), pinocytosis and/or dynamin-dependent endocytosis may also contribute significantly to the cytotoxicity of β2-m amyloid fibrils.

We demonstrated that β2-m amyloid fibrils induce both necrotic and apoptotic changes of HIG-82 cells (Figs 1–3). The necrotic pathway seems to contribute predominantly to the cytotoxicity because the percentage of apoptotic cells induced by β2-m amyloid fibrils is very low (less than several percent) as measured by TUNEL assay (Fig 3). Hotchkiss et al [48] indicated that depending on the injury and the type of cell, a particular pathway of cell death (i.e., apoptosis, autophagy, or necrosis) may predominate and these pathways intertwine with each other at multiple levels. Our observations seem to be compatible with this emerging concept. Interestingly, Gharibyan et al [56] reported that oligomers of hen lysozyme induce apoptosis-like cell death of neuroblastoma SH-SY5Y cells, while the fibrils lead to necrosis-like death. They suggested that a continuum of cross-β-sheet-containing amyloid species (from oligomers to fibrils) may cause cell death through both apoptotic and necrotic pathways.

In the present study, β2-m amyloid fibrils added to the medium adhered to cell surfaces (Fig 4), but did not disrupt artificial plasma membranes (Fig 5). On the other hand, many investigators reported that amyloid fibrils and oligomers directly injure plasma membranes, leading to the reduced cellular viability. Xue et al [30] reported that β2-m, lysozyme, and α-synuclein amyloid fibrils disrupt artificial membranes and reduce cell viability depending on the fibril length. They prepared β2-m amyloid fibrils from wild-type r-β2-m monomers at pH 2.0, while we prepared them from the patient-derived β2-m amyloid fibrils by the repeated extension reaction at pH 7.5 with r-β2-m. This difference in the fibril preparation procedures may

explain the reason why we did not observe the disruption of artificial membranes by β2-m amyloid fibrils. Huang et al [31] reported that both the protofibrils and mature fibrils of lysozyme cause hemolysis and aggregation of erythrocytes. Engel et al [29] indicated that growth of human islet amyloid polypeptide fibrils at the membrane causes membrane leakage. Bucciantini et al [33] reported that the fibrils of yeast prion Sup35p bound to the cell membrane do not penetrate inside the cells, but induce abnormal accumulation and overstabilization of raft domains in the membrane, leading to caspase-8 activation followed by apoptosis of murine endothelioma H-END cells. Malchiodi-Albedi et al [32] reported that the interaction of salmon calcitonin oligomers with plasma membranes increase membrane permeability to Ca^{2+}, resulting in apoptosis of mature neurons. Since we used liposomes composed simply of DMPC and DOPG in the liposome dye release assay (Fig 5), we cannot rule out the possibility that β2-m amyloid fibrils may interact with plasma membrane proteins, sugars, and/or raft domains, leading to the injury of plasma membranes.

Synoviocytes maintain articular tissues by secreting hyaluronic acid into the synovial fluid, removing waste material from the joint cavity by phagocytosis, and regulating the movement of solutes, electrolytes, and proteins between the capillaries and the synovial fluid [54, 55, 57]. Therefore, the injury and dysfunction of synoviocytes induced by β2-m amyloid fibrils may have a pivotal role in the joint destruction in DRA. HIG-82 cells can be activated by the endocytosis of latex beads and secrete collagenase, gelatinase, caseinase, and prostaglandin E_2 [41]. More generally, in inflammatory synovitis, synovial fibroblasts are the principal cells mediating joint destruction by secreting metalloproteinases and many cytokines/chemokines [58]. Thus, in addition to the injury and necrosis/apoptosis of synoviocytes caused by β2-m amyloid fibrils, the activation of synoviocytes by β2-m fibrils followed by the secretion of metalloproteinases and cytokines may also contribute to the pathogenesis of DRA. Future studies are essential to elucidate the mechanism of synoviocyte activation and release of metalloproteinases/cytokines.

In conclusion, we proposed a novel mechanism on the cytotoxicity of β2-m amyloid fibrils in which endocytosed amyloid fibrils may induce necrosis and apoptosis of synoviocytes by disrupting endosomal/lysosomal membranes. The present study gives important insight into not only the pathogenesis of joint destruction in DRA, but also the general mechanism of the cytotoxicity of amyloid fibrils which could be applied to other types of human amyloidoses.

Supporting Information

S1 Fig. Electron micrographs of the fibril preparation prior to (A) and after sonication (B). Samples were spread on carbon-coated grids and negatively stained with 1% phosphotungstic acid (pH 7.0). The images were digitally taken with Hitachi H-7650 transmission electron microscope with an acceleration voltage of 80 kV. The scale bars are 200 nm long.
(TIF)

S2 Fig. Size-exclusion chromatography analysis of the supernatant of the fibril preparation added to the cells. Aliquot of the final fibril preparation was centrifuged at 15 000 rpm for 90 min. A hundred microliters of the supernatant was then applied onto a Superose 12 10/300GL gel-filtration column (GE healthcare, Tokyo, Japan) equilibrated with PBS (-) at 15°C, using a flow rate of 0.5 ml/min while monitoring the absorbance at 280 nm. As a control, 100 μg/ml β2-m monomer solution with 150 mM NaCl was applied.
(TIF)

S3 Fig. Images of the phase contrast microscopy of HIG-82 cells treated with vehicle buffer (A), 100 μg/ml β2-m monomer (B) or amyloid fibrils (C) for 2 days as described in

Materials and Methods. The original magnification was x100.
(TIF)

S4 Fig. The supernatant of the fibril preparation does not affect cellular viability of HIG-82 cells. After HIG-82 cells were incubated for 2 days with Ham's F12 medium containing vehicle buffer, 100 μg/ml β2-m fibrils or the same volume of the supernatant of the fibril preparation, LDH releasing assay (A) and MTT reduction assay (B) were performed as described in Materials and Methods. Data normalized to positive control and vehicle in LDH releasing assay and MTT reduction assay, respectively were presented as mean ± SD of three independent experiments. Statistical analysis was performed by Student's unpaired t-test. $^{*}P < 0.01$, $^{**}P < 0.001$.
(TIF)

S5 Fig. Images of the electron microscopy of HIG-82 cells treated with 100 μg/ml β2-m amyloid fibrils for 6 (A) and 24 hrs (B) after pretreated with cytochalasin D as described in Materials and Methods. The scale bars are 2 μm long.
(TIF)

Acknowledgments

The authors thank Mr. Hitoshi Takagi, Ms. Junko Yamamoto, Ms. Ryo Nomura, Mr. Nobuo Takimoto, Ms. Ai Fujimoto and Ms. Hiromi Okada for excellent technical assistance.

Author Contributions

Conceived and designed the experiments: TO IY DO KH HN. Performed the experiments: TO DO KH. Analyzed the data: TO IY DO KH HN. Wrote the paper: TO HN.

References

1. Koch KM. Dialysis-related amyloidosis. Kidney Int. 1992; 41: 1416–1429. PMID: 1614057
2. Floege J, Ehlerding G. β2-microglobulin-associated amyloidosis. Nephron. 1996; 72: 9–26. PMID: 8903856
3. Floege J, Ketteler M. β2-microglobulin-derived amyloidosis: an update. Kidney Int Suppl. 2001; 78: S164–171. PMID: 11169004
4. Charra B, Calemard E, Uzan M, Terrat JC, Vanel T, Laurent G. Carpal tunnel syndrome, shoulder pain and amyloid deposits in long-term haemodialysis patients. Proc Eur Dial Transplant Assoc Eur Ren Assoc. 1985; 21: 291–295. PMID: 3991513
5. Gejyo F, Arakawa M. Dialysis amyloidosis: current disease concepts and new perspectives for its treatment. Contrib Nephrol. 1990; 78: 47–60. PMID: 2225843
6. Gejyo F, Yamada T, Odani S, Nakagawa Y, Arakawa M, Kunitomo T, et al. A new form of amyloid protein associated with chronic hemodialysis was identified as β2-microglobulin. Biochem Biophys Res Commun. 1985; 129: 701–706. PMID: 3893430
7. Gorevic PD, Munoz PC, Casey TT, DiRaimondo CR, Stone WJ, Prelli FC, et al. Polymerization of intact β2-microglobulin in tissue causes amyloidosis in patients on chronic hemodialysis. Proc Natl Acad Sci U S A. 1986; 83: 7908–7912. PMID: 3532124
8. Campistol JM, Solé M, Bombi JA, Rodriguez R, Mirapeix E, Muñoz-Gomez J, et al. In vitro spontaneous synthesis of β2-microglobulin amyloid fibrils in peripheral blood mononuclear cell culture. Am J Pathol. 1992; 141: 241–247. PMID: 1632465
9. Campistol JM, Bernard D, Papastoitsis G, Solé M, Kasirsky J, Skinner M. Polymerization of normal and intact β2-microglobulin as the amyloidogenic protein in dialysis-amyloidosis. Kidney Int. 1996; 50: 1262–1267. PMID: 8887286
10. Gejyo F, Homma N, Suzuki Y, Arakawa M. Serum levels of β2-microglobulin as a new form of amyloid protein in patients undergoing long-term hemodialysis. N Engl J Med. 1986; 314: 585–586. PMID: 3080684
11. Küchle C, Fricke H, Held E, Schiffl H. High-flux hemodialysis postpones clinical manifestation of dialysis-related amyloidosis. Am J Nephrol. 1996; 16: 484–488. PMID: 8955759

12. van Ypersele de Strihou C, Jadoul M, Malghem J, Maldague B, Jamart J. Effect of dialysis membrane and patient's age on signs of dialysis-related amyloidosis. The Working Party on Dialysis Amyloidosis. Kidney Int. 1991; 39: 1012–1019. PMID: 2067196

13. Davison AM. β2-microglobulin and amyloidosis: who is at risk? Nephrol Dial Transplant. 1995; 10 (Suppl 10): 48–51. PMID: 8825433

14. Lonnemann G, Koch KM. β2-microglobulin amyloidosis: effects of ultrapure dialysate and type of dialyzer membrane. J Am Soc Nephrol. 2002; 13(Suppl 1): S72–77. PMID: 11792765

15. Ohashi K, Hara M, Kawai R, Ogura Y, Honda K, Nihei H, et al. Cervical discs are most susceptible to β2-microglobulin amyloid deposition in the vertebral column. Kidney Int. 1992; 41: 1646–1652. PMID: 1501421

16. Garbar C, Jadoul M, Noël H, van Ypersele de Strihou C. Histological characteristics of sternoclavicular β2-microglobulin amyloidosis and clues for its histogenesis. Kidney Int. 1999; 55: 1983–1990. PMID: 10231463

17. Argilés A, García García M, Mourad G. Phagocytosis of dialysis-related amyloid deposits by macrophages. Nephrol Dial Transplant. 2002; 17: 1136–1138. PMID: 12032213

18. García-García M, Argilés A, Gouin-Charnet A, Durfort M, García-Valero J, Mourad G. Impaired lysosomal processing of β2-microglobulin by infiltrating macrophages in dialysis amyloidosis. Kidney Int. 1999; 55: 899–906. PMID: 10027926

19. Naiki H, Nagai Y. Molecular pathogenesis of protein misfolding diseases: pathological molecular environments versus quality control systems against misfolded proteins. J Biochem. 2009; 146: 751–756. doi: 10.1093/jb/mvp119 PMID: 19643812

20. Bellotti V, Chiti F. Amyloidogenesis in its biological environment: challenging a fundamental issue in protein misfolding diseases. Curr Opin Struct Biol. 2008; 18: 771–779. doi: 10.1016/j.sbi.2008.10.001 PMID: 18952166

21. Eichner T, Radford SE. Understanding the complex mechanisms of β2-microglobulin amyloid assembly. FEBS J. 2011; 278: 3868–3883. doi: 10.1111/j.1742-4658.2011.08186.x PMID: 21595827

22. Stefani M, Dobson CM. Protein aggregation and aggregate toxicity: new insights into protein folding, misfolding diseases and biological evolution. J Mol Med. 2003; 81: 678–699. PMID: 12942175

23. Eisenberg D, Jucker M. The amyloid state of proteins in human diseases. Cell. 2012; 148: 1188–1203. doi: 10.1016/j.cell.2012.02.022 PMID: 22424229

24. Cecchi C, Stefani M. The amyloid-cell membrane system. The interplay between the biophysical features of oligomers/fibrils and cell membrane defines amyloid toxicity. Biophys Chem. 2013; 182: 30–43. doi: 10.1016/j.bpc.2013.06.003 PMID: 23820236

25. Haass C, Selkoe DJ. Soluble protein oligomers in neurodegeneration: lessons from the Alzheimer's amyloid -peptide. Nat Rev Mol Cell Biol. 2007; 8: 101–112. PMID: 17245412

26. Okada T, Wakabayashi M, Ikeda K, Matsuzaki K. Formation of toxic fibrils of Alzheimer's amyloid -protein-(1–40) by monosialoganglioside GM1, a neuronal membrane component. J Mol Biol. 2007; 371: 481–489. PMID: 17582434

27. Okada T, Ikeda K, Wakabayashi M, Ogawa M, Matsuzaki K. Formation of toxic A(1–40) fibrils on GM1 ganglioside-containing membranes mimicking lipid rafts: polymorphisms in A(1–40) fibrils. J Mol Biol. 2008; 382: 1066–1074. doi: 10.1016/j.jmb.2008.07.072 PMID: 18692507

28. Leung E, Guo L, Bu J, Maloof M, El Khoury J, Geula C. Microglia activation mediates fibrillary amyloid-β toxicity in the aged primate cortex. Neurobiol Aging. 2011; 32: 387–397. doi: 10.1016/j.neurobiolaging.2009.02.025 PMID: 19349094

29. Engel MF, Khemtémourian L, Kleijer CC, Meeldijk HJ, Jacobs J, Verkleij AJ, et al. Membrane damage by human islet amyloid polypeptide through fibril growth at the membrane. Proc Natl Acad Sci U S A. 2008; 105: 6033–6038. doi: 10.1073/pnas.0708354105 PMID: 18408164

30. Xue WF, Hellewell AL, Gosal WS, Homans SW, Hewitt EW, Radford SE. Fibril fragmentation enhances amyloid cytotoxicity. J Biol Chem. 2009; 284: 34272–34282. doi: 10.1074/jbc.M109.049809 PMID: 19808677

31. Huang B, He J, Ren J, Yan XY, Zeng CM. Cellular membrane disruption by amyloid fibrils involved intermolecular disulfide cross-linking. Biochemistry. 2009; 48: 5794–5800. doi: 10.1021/bi900219c PMID: 19449893

32. Malchiodi-Albedi F, Contrusciere V, Raggi C, Fecchi K, Rainaldi G, Paradisi S, et al. Lipid raft disruption protects mature neurons against amyloid oligomer toxicity. Biochim Biophys Acta. 2010; 1802: 406–415. doi: 10.1016/j.bbadis.2010.01.007 PMID: 20060899

33. Bucciantini M, Nosi D, Forzan M, Russo E, Calamai M, Pieri L, et al. Toxic effects of amyloid fibrils on cell membranes: the importance of ganglioside GM1. FASEB J. 2012; 26: 818–831. doi: 10.1096/fj.11-189381 PMID: 22071505

34. Walsh P, Vanderlee G, Yau J, Campeau J, Sim VL, Yip CM, et al. The mechanism of membrane disruption by cytotoxic amyloid oligomers formed by prion protein(106–126) is dependent on bilayer composition. J Biol Chem. 2014; 289: 10419–10430. doi: 10.1074/jbc.M113.515866 PMID: 24554723

35. Habib LK, Lee MT, Yang J. Inhibitors of catalase-amyloid interactions protect cells from β-amyloid-induced oxidative stress and toxicity. J Biol Chem. 2010; 285: 38933–38943. doi: 10.1074/jbc.M110.132860 PMID: 20923778

36. Demuro A, Parker I. Cytotoxicity of intracellular $A\beta_{42}$ amyloid oligomers involves Ca^{2+} release from the endoplasmic reticulum by stimulated production of inositol trisphosphate. J Neurosci. 2013; 33: 3824–3833. doi: 10.1523/JNEUROSCI.4367-12.2013 PMID: 23447594

37. Meratan AA, Ghasemi A, Nemat-Gorgani M. Membrane integrity and amyloid cytotoxicity: a model study involving mitochondria and lysozyme fibrillation products. J Mol Biol. 2011; 409: 826–838. doi: 10.1016/j.jmb.2011.04.045 PMID: 21565199

38. Porter MY, Routledge KE, Radford SE, Hewitt EW. Characterization of the response of primary cells relevant to dialysis-related amyloidosis to β2-microglobulin monomer and fibrils. PLoS One. 2011; 6: e27353. doi: 10.1371/journal.pone.0027353 PMID: 22096558

39. Sheynis T, Friediger A, Xue WF, Hellewell AL, Tipping KW, Hewitt EW, et al. Aggregation modulators interfere with membrane interactions of β2-microglobulin fibrils. Biophys J. 2013; 105: 745–755. doi: 10.1016/j.bpj.2013.06.015 PMID: 23931322

40. Jakhria T, Hellewell AL, Porter MY, Jackson MP, Tipping KW, Xue WF, et al. β2-Microglobulin amyloid fibrils are nanoparticles that disrupt lysosomal membrane protein trafficking and inhibit protein degradation by lysosomes. J Biol Chem. 2014; 289: 35781–35794. doi: 10.1074/jbc.M114.586222 PMID: 25378395

41. Georgescu HI, Mendelow D, Evans CH. HIG-82: an established cell line from rabbit periarticular soft tissue, which retains the "activatable" phenotype. In Vitro Cell Dev Biol. 1988; 24: 1015–1022. PMID: 2846503

42. Chiba T, Hagihara Y, Higurashi T, Hasegawa K, Naiki H, Goto Y. Amyloid fibril formation in the context of full-length protein: effects of proline mutations on the amyloid fibril formation of β2-microglobulin. J Biol Chem. 2003; 278: 47016–47024. PMID: 12958308

43. Kihara M, Chatani E, Sakai M, Hasegawa K, Naiki H, Goto Y. Seeding-dependent maturation of β2-microglobulin amyloid fibrils at neutral pH. J Biol Chem. 2005; 280: 12012–12018. PMID: 15659393

44. Ookoshi T, Hasegawa K, Ohhashi Y, Kimura H, Takahashi N, Yoshida H, et al. Lysophospholipids induce the nucleation and extension of β2-microglobulin-related amyloid fibrils at a neutral pH. Nephrol Dial Transplant. 2008; 23: 3247–3255. doi: 10.1093/ndt/gfn231 PMID: 18467373

45. Smith PK, Krohn RI, Hermanson GT, Mallia AK, Gartner FH, Provenzano MD, et al. Measurement of protein using bicinchoninic acid. Anal Biochem. 1985; 150: 76–85. PMID: 3843705

46. Wakabayashi M, Matsuzaki K. Formation of amyloids by Aβ-(1–42) on NGF-differentiated PC12 cells: roles of gangliosides and cholesterol. J Mol Biol. 2007; 371: 924–933. PMID: 17597153

47. Kumar V, Abbas AK, Aster JC. Cell injury, cell death, and adaptations. In: Kumar V, Abbas AK, Aster JC, editors. Robbins Basic Pathology, 9th ed. Philadelphia, PA: Saunders, 2012; pp. 1–28.

48. Hotchkiss RS, Strasser A, McDunn JE, Swanson PE. Cell death. N Engl J Med. 2009; 361: 1570–1583. doi: 10.1056/NEJMra0901217 PMID: 19828534

49. Davies P, Allison AC. Effects of cytochalasin B on endocytosis and exocytosis. Front Biol. 1978; 46: 143–160. PMID: 352738

50. Cooper JA. Effects of cytochalasin and phalloidin on actin. J Cell Biol. 1987; 105: 1473–1478. PMID: 3312229

51. Ditaranto K, Tekirian TL, Yang AJ. Lysosomal membrane damage in soluble Aβ-mediated cell death in Alzheimer's disease. Neurobiol Dis. 2001; 8: 19–31. PMID: 11162237

52. Umeda T, Tomiyama T, Sakama N, Tanaka S, Lambert MP, Klein WL, et al. Intraneuronal amyloid β oligomers cause cell death via endoplasmic reticulum stress, endosomal/lysosomal leakage, and mitochondrial dysfunction in vivo. J Neurosci Res. 2011; 89: 1031–1042. doi: 10.1002/jnr.22640 PMID: 21488093

53. Guan J, Mishra S, Qiu Y, Shi J, Trudeau K, Las G, et al. Lysosomal dysfunction and impaired autophagy underlie the pathogenesis of amyloidogenic light chain-mediated cardiotoxicity. EMBO Mol Med. 2014; 6: 1493–1507. doi: 10.15252/emmm.201404190 PMID: 25319546

54. Barland P, Novikoff AB, Hamerman D. Electron microscopy of the human synovial membrane. J Cell Biol. 1962; 14: 207–220. PMID: 13865038

55. Fell HB. Synoviocytes. J Clin Pathol Suppl. 1978; 12: 14–24.

56. Gharibyan AL, Zamotin V, Yanamandra K, Moskaleva OS, Margulis BA, Kostanyan IA, et al. Lysozyme amyloid oligomers and fibrils induce cellular death via different apoptotic/necrotic pathways. J Mol Biol. 2007; 365: 1337–1349. PMID: 17134716

57. Bullough PG. Joints. In: Mills SE, editor. Histology for Pathologists, 4th ed. Philadelphia, PA: Lippincott Williams & Wilkins; 2012. pp. 107–130.

58. Ritchlin C. Fibroblast biology. Effector signals released by the synovial fibroblast in arthritis. Arthritis Res. 2000; 2: 356–360. PMID: 11094448
